# Entropy, Irreversibility, and Time-Series Deep Learning of Kinematic and Kinetic Data for Gait Classification in Children with Cerebral Palsy, Idiopathic Toe Walking, and Hereditary Spastic Paraplegia

**DOI:** 10.3390/s25134235

**Published:** 2025-07-07

**Authors:** Alfonso de Gorostegui, Massimiliano Zanin, Juan-Andrés Martín-Gonzalo, Javier López-López, David Gómez-Andrés, Damien Kiernan, Estrella Rausell

**Affiliations:** 1 PhD Program in Neuroscience, Universidad Autonoma de Madrid-Cajal Institute, 28029 Madrid, Spain; alfonso.degorostegui@gmail.com; 2 Department of Anatomy, Histology & Neuroscience, School of Medicine, Universidad Autónoma de Madrid (UAM), 28029 Madrid, Spain; estrella.rausell@uam.es; 3 Instituto de Física Interdisciplinar y Sistemas Complejos IFISC (CSIC-UIB), Campus UIB, 07122 Palma de Mallorca, Spain; 4 Escuela Universitaria de Fisioterapia de la ONCE, Universidad Autónoma de Madrid, 28034 Madrid, Spain; jumago@once.es; 5 Department of Rehabilitation, Hospital Universitario Infanta Sofía, 28702 Madrid, Spain; jlopezlo@salud.madrid.org; 6 Fundación para la Investigación e Innovación Biomédica del Hospital Universitario Infanta Sofía y Hospital del Henares, 28702 Madrid, Spain; 7 Departamento de Medicina, Salud y Deporte, Universidad Europea de Madrid, Villaviciosa de Odón, 28670 Madrid, Spain; 8 Pediatric Neurology, ERN-RND, Euro-NMD, Vall d’Hebron Institut de Recerca (VHIR), Hospital Universitari Vall d’Hebron, 08035 Barcelona, Spain; 9 Movement Analysis Laboratory, Central Remedial Clinic, Clontarf, D03 R973 Dublin, Ireland; dkiernan@crc.ie

**Keywords:** cerebral palsy, idiopathic toe walking, hereditary spastic paraplegia, deep learning, entropy, time irreversibility

## Abstract

The use of gait analysis to differentiate among paediatric populations with neurological and developmental conditions such as idiopathic toe walking (ITW), cerebral palsy (CP), and hereditary spastic paraplegia (HSP) remains challenging due to the insufficient precision of current diagnostic approaches, leading in some cases to misdiagnosis. Existing methods often isolate the analysis of gait variables, overlooking the whole complexity of biomechanical patterns and variations in motor control strategies. While previous studies have explored the use of statistical physics principles for the analysis of impaired gait patterns, gaps remain in integrating both kinematic and kinetic information or benchmarking these approaches against Deep Learning models. This study evaluates the robustness of statistical physics metrics in differentiating between normal and abnormal gait patterns and quantifies how the data source affects model performance. The analysis was conducted using gait data sets from two research institutions in Madrid and Dublin, with a total of 81 children with ITW, 300 with CP, 20 with HSP, and 127 typically developing children as controls. From each kinematic and kinetic time series, Shannon’s entropy, permutation entropy, weighted permutation entropy, and time irreversibility metrics were derived and used with Random Forest models. The classification accuracy of these features was compared to a ResNet Deep Learning model. Further analyses explored the effects of inter-laboratory comparisons and the spatiotemporal resolution of time series on classification performance and evaluated the impact of age and walking speed with linear mixed models. The results revealed that statistical physics metrics were able to differentiate among impaired gait patterns, achieving classification scores comparable to ResNet. The effects of walking speed and age on gait predictability and temporal organisation were observed as disease-specific patterns. However, performance differences across laboratories limit the generalisation of the trained models. These findings highlight the value of statistical physics metrics in the classification of children with different toe walking conditions and point towards the need of multimetric integration to improve diagnostic accuracy and gain a more comprehensive understanding of gait disorders.

## 1. Introduction

Toe walking is defined as the absence of heel strike during the stance phase of the gait cycle [1]. While it can appear in typically developing children, a persistent toe walking pattern after the age of 3 years can be considered abnormal [2] and may be associated with other underlying conditions, such as cerebral palsy (CP) [3,4], or rare progressive neurogenetic problems, such as hereditary spastic paraplegias (HSPs) [5]. If all primary causes are reasonably excluded after careful evaluation, the condition is diagnosed as idiopathic toe walking (ITW) [6]. The differential diagnosis of toe walking is based on an adequate clinical evaluation and on the use of magnetic resonance imaging to look for central nervous system insult [7] or genetic testing for HSPs and other rare neurogenetic disorders [8]. Understanding how toe walking emerges from childhood disorders with very different primary causes is a relevant scientific endeavour, as it could allow us to gain insights about the mechanisms used by the central nervous system to ensure gait control, enhance clinical evaluation in challenging cases, and develop strategies that could augment therapeutic efficacy and monitoring.

A popular method to quantify gait alterations is the instrumented gait analysis (IGA), i.e., a suite of objective techniques used to quantitatively assess human walking patterns [9]. These techniques are increasingly adopted in both clinical and research settings, enabling the identification of patient-specific gait abnormalities by capturing whole-body movement and extracting numerous spatiotemporal parameters—such as walking speed and step length. Additionally, IGA provides high-frequency kinematic and kinetic data from the joints that coordinate the lower limb segments throughout the gait cycle. Numerous applications of IGA can be found in the literature, specifically tackling conditions like ITW [10,11,12,13,14,15], CP [16,17,18], and HSP [19,20,21]. At the same time, fewer studies have analysed these data as emergent results of complex dynamics in which neural control needs to interact with locomotor systems—something common in the case of other physiological signals [22,23,24,25]. Examples include the use of concepts like entropy [26,27,28], multi-scale entropy [29], fractal analysis [30], and time irreversibility [31,32]. Therein, the main objective has usually been the identification of features that change in a given pathology with respect to control subjects, thus potentially yielding a biomarker of the condition. Still, these concepts have mostly been used in an independent fashion, thus providing little insights on the underlying mechanisms of gait control and their alterations.

In this paper, we aim to apply a variety of metrics drawn from statistical physics to estimate some of these properties [33]. We will compare several groups (healthy children, children with CP, children with HSP, and children with ITW), recruited in two different gait laboratories, by applying several methods to calculate the entropy and irreversibility of the corresponding gait signals. Entropies are estimators of the uncertainty in (or the unpredictability of) time series, hence in the gait system; we then hypothesise that changes in them could reflect different motor control strategies from neural commands. Time irreversibility quantifies the lack of temporal symmetry of the signal and, among others, is the result of how much it depends on its history, like the effect of an accumulated memory in the current performance of the system. In terms of gait motor control, the loss of irreversibility could be interpreted as the loss of the required coordination that supervises the correct sequencing of gait events.

We will further benchmark the discrimination capability of these statistical physics metrics against that of Deep Learning (DL) models, i.e., state-of-the-art classification models inspired by the way human neurons are organised. These models construct their own internal representation of the data and thus do not require any pre hoc feature extraction. By comparing the results yielded by both approaches, we will quantify their capacity to distinguish between groups based on raw gait signals, hence their relevance in a clinical context. More importantly, these results will also yield information about the underlying mechanisms used by the central nervous system to maintain gait control in the presence of pathologies.

Overall, to the best of our knowledge, this is the first study that combines the use of DL with the entropy and irreversibility of gait signals in different toe walking groups. While other studies have focused on DL [34,35,36] or complexity metrics alone [37,38], this study incorporates both approximations in order to identify mechanisms and markers that differentiate between the neurological (CP and HSP) and non-neurological (ITW) toe walking origin. Furthermore, having data collected from two different laboratories allows us to determine the generalisability of our results across different conditions and patient characteristics. This approach not only increases the confidence in our conclusions but also contributes to providing objective and more reliable diagnostic tools.

## 2. Methods

### 2.1. Participants

A total of 528 children from two laboratories (454 from Dublin and 74 from Madrid, 127—24%—of which were healthy volunteers) participated in the study. Table 1 shows a description of their clinical and spatiotemporal features. Data from a total of 2670 gait cycles were used in the study.

#### 2.1.1. Movement Analysis Laboratory, CRC, Dublin

A group of 96 healthy children (4–16 years) were selected as the control normal reference sample. At the time of the assessment, no walking impairment was detected. Two groups of children with gait abnormalities were identified: (1) 300 children (4–16 years) clinically diagnosed with bilateral spastic CP and (2) 58 children (4–16 years) with ITW. The Central Remedial Clinic (CRC) is a national referral centre for children and adults with disability in the Republic of Ireland. The local Ethics Committee approved this study (CRC Ethics approval 12116, 28 March 2022).

The age distribution was similar across all groups, with no significant mean differences. However, the sex distribution in idiopathic toe walkers was unbalanced, reporting more male patients (75.9%) than the control (58%) and CP (50%) groups. Also note that the sample size was different between the study groups due to the limited availability of idiopathic toe walkers that fulfilled the inclusion criteria.

#### 2.1.2. Laboratory of Human Movement, ONCE-UAM, Madrid

A group of 31 healthy children (4–16 years) were recruited and used as the control normal reference sample. At the time of the assessment, no walking impairment was detected. Two groups of children with gait abnormalities were identified: (1) idiopathic toe walkers and (2) individuals clinically diagnosed with HSP. The groups were compared against the reference category. Instrumented gait analysis (IGA) data were collected from 23 children (4–16 years) with ITW and 20 children (4–16 years) with HSP. Both groups were recruited in reference hospitals in Madrid serving the Madrid region but also other Spanish areas in the case of HSP.

The control subjects had similar sex and age distributions to the groups with walking impairments. Note that the groups of patients did not match in number due to the limited availability of participants that fulfilled the inclusion criteria. The local Ethics Committee approved this study (Comité de Ética de la Investigación, Universidad Autónoma de Madrid, CEI-27-685, 6 April 2011), and individuals were all subjected to examination after giving informed written consent. The work was carried out in accordance with the Code of Ethics of the World Medical Association (Declaration of Helsinki).

**Table 1:** Clinical and spatiotemporal gait features of subjects from Dublin and Madrid labs. Normalised walking speed is calculated as ratio between speed measured by IGA and length of the limb. *GMFCS*: Gross Motor Function Classification System.

		**Dublin**					**Madrid**		
	**Control**	**ITW**	**CP**	***p*** **-Value**		**Control**	**ITW**	**HSP**	***p*** **-Value**
	**(N = 96)**	**(N = 58)**	**(N = 300)**			**(N = 31)**	**(N = 23)**	**(N = 20)**	
**Sex** (n)									
F	48 (50%)	14 (24.1%)	126 (42%)	0.006		12 (38.7%)	9 (39.1%)	7 (35%)	0.954
M	48 (50%)	44 (75.9%)	174 (58%)			19 (61.3%)	14 (60.9%)	13 (65%)	
**Age** (years)									
Median [Min, Max]	9.00 [4.00, 16.0]	9.09 [4.00, 15.0]	9.65 [4.00, 16.0]	0.331		9.00 [5.00, 16.0]	9.00 [4.00, 12.0]	6.50 [4.00, 16.0]	0.23
**Weight** (kg)									
Median [Min, Max]	31.3 [14.6, 94.7]	35.3 [19.5, 76.7]	31.9 [14.4, 103]	0.574		28.6 [18.5, 75.3]	37.0 [16.0, 55.5]	27.1 [15.9, 64.0]	0.651
**Height** (cm)									
Median [Min, Max]	1.37 [1.05, 1.83]	1.41 [1.12, 1.79]	1.35 [1.02, 1.80]	0.266		1.38 [1.00, 1.82]	1.35 [1.08, 1.60]	1.22 [1.05, 1.65]	0.119
**GMFCS**									
I			132 (44%)					8 (40%)	
II			90 (30%)					11 (55%)	
III			10 (3.3%)					1 (5%)	
Missing			68 (22.7%)						
**Normalised walking speed** (s^−1^)									
Median [Min, Max]	1.74 [1.16, 2.44]	1.67 [1.07, 2.53]	1.48 [0.642, 2.75]	0.001		1.49 [1.15, 2.03]	1.22 [0.933, 1.84]	1.48 [0.671, 1.98]	0.021
**Cadence** (steps/s)									
Median [Min, Max]	2.18 [1.70, 2.95]	2.10 [1.66, 2.96]	2.10 [1.16, 3.32]	0.004		1.92 [1.56, 2.73]	1.94 [1.42, 2.47]	2.06 [1.59, 2.57]	0.388
**Stance time** (% gait cycle)									
Median [Min, Max]	61.2 [57.8, 66.7]	61.6 [57.5, 66.4]	63.7 [56.8, 74.0]	0.001		63.3 [60.2, 66.8]	64.2 [61.7, 68.2]	65.8 [62.7, 70.6]	0.001

### 2.2. Gait Data Collection and Data Preprocessing

Both laboratories employed a CODA cx1 active marker system (Codamotion Ltd., Leicestershire, UK) with embedded multi-component Kistler force platforms (Kistler Instrument Corporation, Amherst, NY, USA). Kinematic data were captured at 200 Hz with markers placed on the lower limbs according to a modified Helen Hayes model [39,40]. Kinetic data were captured at 1000 Hz in Madrid and at 400 Hz in Dublin. Some differences in equipment setup were observed between laboratories, specifically in the number of cameras and the models of the force platforms used. Children were instructed to walk several times from one end to the other of a long walkway path at their natural, spontaneous speed. They were encouraged to perform the task without support. For those children who required it, assistance was provided by their parents, who supported the children by holding their hands in front of them. Parents were allowed to guide the child and partially support the child’s weight. The system acquired continuous real-time kinematic and kinetic data during each complete walk on the walkway. After the acquisition session, individual gait cycles were isolated by manually marking their beginning (heel contact) and their end (subsequent heel strike of the same foot). Cycles were then reviewed to select those in which gait was stable, which usually coincided with those obtained from the central meters of the walkway. Next, each selected cycle was again reviewed to check the consistency of signal repetition. The whole post-acquisition selection process was performed by two independent reviewers, with the help of custom software programmed in R. Such program is designed to detect abnormalities in cycle marking or signal reception, eliminate outliers in discrete kinematic and kinetic parameters that might arise from marker failure or displacement, and automate data extraction. This data validation process resulted in 2–5 valid cycles from each side (left or right leg of the children). We analysed the kinematic time series of 5 joints (pelvis, hip, knee, ankle, and foot) in the sagittal, horizontal, and coronal (or frontal) planes for each gait cycle. This comprised approximately 200 time epochs per cycle. Additionally, we examined the kinetic time series of the ground reaction vector’s three components (sagittal, anteroposterior, and mediolateral), as well as the moments and powers of three joints (hip, knee, and ankle). For the sake of conciseness, these will respectively be denoted as GRF (ground reaction force) and M&P (moments and powers) in what follows.

### 2.3. Statistical Physics Metrics

The previously described gait time series were analysed using four metrics drawn from statistical physics theory. These were chosen because they describe different dynamical aspects, which can then be interpreted from a biological viewpoint [33]. A synthesis of the main concepts is included in Table 2, while a detailed description is reported below:

- *Shannon’s entropy* (SE): It is a fundamental entropy metric describing the uncertainty associated with the amplitude of the values in the time series. It is defined as $E=-\sum_{i}p\left(i\right)\ln p\left(i\right)$, where p(i) represents the probability of observing the *i*-th symbol. In order to convert the original time series into a sequence of discrete symbols, values are transformed according to a number of identically sized bins given by the square root of the length of the time series. Values of *E* approaching zero indicate no uncertainty in the time series and thus that all values are the same; on the other hand, an entropy close to the maximum ($E_{\max}=\ln\left|i\right|$) suggests a uniform distribution of values.
- *Permutation entropy* (PE): It is Shannon’s entropy calculated over the distribution of ordinal patterns [41]. Given a time series, sub-windows of size *D* (also called the embedding dimension) are extracted; then, each sub-window is associated to a symbol representing the permutation that has to be performed in order to sort its values. Finally, Shannon’s entropy is calculated on the appearance frequency of the D! possible ordinal patterns; in mathematical terms, this is calculated as $PE=-\sum_{i}p\left(\pi_{i}\right)\ln p\left(\pi_{i}\right)$, where $p(\pi_{i})$ is the appearance probability of the ordinal pattern $\pi_{i}$. Compared with the previous entropy, which is only sensitive to the distribution of individual values, permutation entropy is able to describe the causal relations between neighbouring values and has proven effective in describing multiple types of dynamical systems [42,43,44]. Due to the limited length of the time series available in this study and in order to obtain statistically significant distributions, *D* was here set to four [45].
- *Weighted permutation entropy*: It is the variation in the aforementioned permutation entropy, in which the contribution of each sub-window is weighted according to the variance of its values [46,47]. In mathematical terms, the previous probability $p(\pi_{i})$ is substituted by a weighted version $p_{w}\left(\pi_{i}\right)$; the latter includes a weighting proportional to the amplitude of the signal, i.e., $p_{w}\left(\pi_{i}\right)=w_{\pi_{i}}p\left(\pi_{i}\right)$ with $w_{\pi_{i}}=1/D\sum_{k=1}^{D}{\left(x_{k}-\bar{x}\right)}^{2}$. In other words, the contribution of each pattern includes both the frequency at which it appears, as well as how much its values deviate from the average. This ensures that the patterns of very small amplitude, e.g., only caused by noise, will have a smaller impact on the final metric. Its main advantage resides in the fact that it encodes information beyond the order structure of the time series.
- *Irreversibility*: It is a family of metrics describing the time-reversal invariance of a time series, i.e., the fact that a time series may be recognisable, in a statistical sense, from its time-reversal counterpart. To illustrate, a movie showing ice cubes melting in a glass is irreversible, as it is possible to detect whether it is played forward or backward. In the context of time-series analysis, irreversibility means that a given metric is different when calculated over the original series and over the backward version of the same. Irreversibility is usually associated with non-linear dynamics, (linear and non-linear) non-Gaussian dynamics, and the presence of memory [48,49,50]. When applied to gait, it implies that the dynamics is the result of its history and hence that some delayed control is in place. Several irreversibility metrics have been tested, specifically the BDS [51,52], Ramsey [53], and DFK [54] tests; the local Clustering Coefficient (lCC) [55]; the Ternary Coding method [56]; and an ensemble approach—for further information about them, the interested reader is referred to [57,58] and the references therein. Of these, the BDS test [51,52] was chosen, as it yielded consistently good results—see Figure A5.

### 2.4. Linear Mixed Effect Models

Linear mixed effects models were employed to examine the influence of age, walking speed, and disease condition on statistical physics metrics, with healthy individuals serving as the reference group. These models allowed for the consideration of both fixed effects (age, normalised walking speed, and disease condition) and random effects (individual differences) while accommodating the nested structure of the data (side within individuals). Additionally, interactions between disease conditions and both age and walking speed were included to elucidate potential moderating effects. This approach facilitated a comprehensive understanding of how these factors individually and interactively contribute to variations in the studied physics metrics, thus providing insights into the complex interplay between physiological parameters and disease status. We used Bayesian mixed effects models for continuous variables programmed with non-informative priors using the MCMCglmm package in R. We used 2,300,000 iterations with a burn-in of 300,000 and a thinning of 100 in the Markov chain Monte Carlo algorithm. We calculated the posterior coefficient beta for each fixed term and its 95% highest posterior interval as the credible interval. In order to provide a graphical representation of the results, we standardised these coefficients with the pooled standard deviation for each statistical physics metric and showed the results in Forest plots.

### 2.5. Classification and Validation

Once the statistical physics metrics had been extracted from each time series, we evaluated their usefulness in discriminating between the groups by means of a classification task. Classification is important both for demonstrating the presence of a particular pattern in statistical physics metrics in each disease and for the clinical correlation of the statistical physics metrics and gait patterns. Specifically, for each of the six pairs of groups (control vs. ITW, control vs. CP, and ITW vs. CP for the Dublin data set; and control vs. ITW, control vs. HSP, and ITW vs. HSP for the Madrilenian data set), we used a Random Forest model [59] (with 500 trees; scikit-learn Python implementation [60]) to predict the subjects’ class and evaluated the result by the corresponding precision (i.e., the fraction of subjects correctly classified). In order to better represent the generalisability of such models, given the reduced number of subjects, we used leave-one-out cross-validation, in which the model was iteratively tested on the information of one subject and was trained with the information of all other ones. The final classification score was given by the average precision obtained in 100 independent realisations of the task. As a last note and in order to avoid biased results due to the fact that the groups were not of the same size, in each task, a random subset of subjects of the largest group were sampled to match the size of the smallest group. In synthesis, the result of the classification score was a number between 0.5 and 1, representing situations in which the model performed uninformed and perfect classification, respectively, hence situations in which the analysed features encode no or full information about the differences between the two groups, respectively.

### 2.6. Deep Learning Classification

In parallel with the previous analysis, the original gait time series were also classified using a Deep Learning model. Deep Learning is a paradigm for designing machine learning models inspired by the way the human brain processes information, which yields unparalleled results in many classification tasks and data analysis at large. On the other hand, Deep Learning models are usually black boxes, meaning that it is not easy to explain how and why a given solution is yielded. This latter point is not a problem in the context of this work, as such models are used to provide a reference value against which the results yielded by the statistical physics metrics are compared, or in other words, to understand how close the latter ones are to providing perfect information about the data.

The Deep Learning model here considered is Residual Network (ResNet) [61], for being one of most effective approaches (if not the most effective) in the classification of time series [62,63]. ResNets are composed of 11 layers, with the first 9 of them being convolutional, followed by a global average pooling layer that averages the time series across the time dimension and a final *softmax* classifier. The used implementation corresponds to a custom one based on Python and the Tensorflow [64] and Keras [65] libraries; training and evaluation were performed on a cluster of machines with AMD 7282 processors with 16 cores at 2.8 GHz and NVIDIA RTX 3090 GPUs.

## 3. Results

### 3.1. Description of Distribution of Statistical Physics Metrics Within and Between Groups of Gait Disorders

For the kinematic time series, permutation entropy differs between planes of movement and cohorts (Figure A18 and Figure A19). Movements in the adduction–abduction and rotational planes tend to have greater permutation entropy values than flexion–extension, and in general, the permutation entropy of patients is higher than that of control subjects for both laboratories. On the other hand, Shannon’s entropy reveals that for every kinematic time series, almost identical median values are shared between groups (Figure A20 and Figure A21). The only differences arise in the comparison between labs: while the Dublin distributions are more narrow and centred around the median, in the Madrilenian lab, they are more dispersed towards lower values. Differences in irreversibility (Figure A22 and Figure A23) can be observed in the flexion–extension and rotational movements of the ankle. Healthy subjects have slightly more irreversible values at the median level, but in ankle rotation, some patients reach more extreme (irreversible) levels.

For the GRF time series (Figure A24 and Figure A25), permutation entropy varies according to the movement plane in healthy subjects and patients. The mediolateral shear force tends to have greater values than the sagittal or anteroposterior components. This is true for the cohorts of both laboratories, where a similar pattern can be observed, although results are, in general, higher in the Madrilenian data set. Nevertheless, there is no substantial difference between healthy and impaired median values. In the case of Shannon’s entropy, similar distributions can be observed between force components, labs, and groups, except for CP patients. In the mediolateral, anteroposterior, and sagittal planes, the CP Shannon’s entropy almost exclusively populates the extremely distributed values, possibly reflecting the heterogeneity of the condition. This is further observed in the BDS irreversibility distribution, where CP in the sagittal plane and both CP and ITW in the mediolateral plane have more negative (more irreversible) values than healthy subjects.

In Figure A26 and Figure A27, the statistical physics of the flexion moments are depicted for both laboratories. Across all joints, the permutation entropy values are higher for the hip and similarly distributed between the knee and ankle for every group. However, for both laboratories, the control group presents a lower median value than the rest of the conditions, while similar values are observed between the impaired cohorts. Regarding Shannon’s entropy, the ankle flexion moment has the lowest median values, followed by the knee and the hip. In the Dublin cohorts, there are no differences between the groups’ median values at each joint, but the Madrilenian healthy group showed the lowest Shannon’s entropy values for the hip moment and the highest for the knee moment. From the BDS irreversibility distributions, it can be observed that both knee and ankle moments reach the maximal values while the hip is at the lowest value, with this being consistent for all groups and laboratories. Finally, Figure A28 and Figure A29 represent the distributions of the entropy and irreversibility metrics of flexion powers in the Dublin and Madrilenian cohorts, respectively. In both cases, the permutation entropy values do not exhibit significant differences across joint powers and conditions. This is different, though, for Shannon’s entropy, where in both labs, the hip flexion power reaches the maximum level, followed by the knee and ankle flexion powers. Interestingly, the differences between groups are subject to the chosen joint power; for the hip and ankle, the median values are almost identical, with the exception of the controls from Dublin, who show lower values; and for the knee, they are increased in healthy subjects compared with the rest of the conditions. The changes are less noticeable, however, in BDS irreversibility, where the median values are all close to 0; differences in the distribution shapes still indicate more irreversibility in the knee flexion power.

### 3.2. Comparison of Statistical Physics Metrics Between Groups of Gait Disorders

In the Dublin lab, the analysis with linear mixed models reveals specific effects of age, walking speed, and condition (CP and ITW) on gait entropies and time irreversibility metrics (Figure A30, Figure A31 and Figure A32). Age exhibits varying but small-sized effects on permutation entropy (PE), Shannon’s entropy (SE), and BDS irreversibility, generally influencing parameters in flexion–extension movements such as ankle flexion. Additionally, walking speed demonstrates significant patterns of negative correlation with permutation entropy values in both kinematic and kinetic variables; positive correlations with the Shannon’s entropy values of some kinematic time series for all joints; and a combination of both negative and positive correlations with BDS irreversibility. When each condition (CP and ITW) is considered alone, significant differences can be observed with respect to the healthy controls. In CP, the gait entropy values are significantly different in the kinematics of the ankle and knee and at all force levels, while irreversibility changes appear in the kinematics of proximal joints and in the flexion moments of the ankle and the knee. However, if the patient suffers from ITW, each metric provides a different response. Entropy patterns are not maintained, and changes are observed in the levels of both permutation and Shannon’s entropies, but only for the kinematics of distal joints, such as the ankle and the forefoot; large-sized effects are observed in the irreversibility of specific features such as pelvic flexion, ankle rotation, and knee and ankle flexion moments. On the other hand, interaction terms of the condition with age and speed reveal unique effects on gait dynamics. Interactions between age and CP tend to intensify the effects seen for age and condition alone on permutation entropy, Shannon’s entropy, and BDS values. For CP, adding speed to the condition emphasises the differences in flexion–extension movements captured by both entropies. It also highlights both hip and pelvic dynamics measured by irreversibility and reveals new differential patterns of forefoot flexion due to the interaction of velocity and condition. When ITW is considered instead, the interaction with age removes the significant variables that were observed in the condition alone, reaching entropy and irreversibility levels similar to those of healthy children. For ITW, increasing speed mainly affects the permutation entropy and Shannon’s entropy values for ankle flexion and its flexion moment, while BDS maintains the differential effects found for pelvic flexion, ankle rotation and knee flexion moment.

In the Madrilenian lab, the effects of age, walking speed and condition on gait entropies and irreversibility reveal differences between healthy children and those with ITW or HSP (Figure A33, Figure A34 and Figure A35). It can be observed that age predominantly influences the permutation entropy, Shannon’s entropy, and BDS irreversibility values for joint moments and powers. However, the effect of increasing speed shows significant alterations in joint kinematics, particularly affecting hip flexion and rotation, knee and forefoot movements, and to a lesser extent, the flexion powers of the hip and the knee. Differences from the healthy controls are seen in the effects of suffering from a condition (HSP and ITW). For HSP, when considered alone, significant changes are observed in the Shannon’s entropy values of the pelvis and in the BDS value of ankle flexion, whereas for ITW, gait differences are only captured in BDS, revealing changes in pelvic flexion, mediolateral shear force, and hip power. When the interactions between the condition and both age and speed are considered, unique patterns are observed in the permutation entropy, Shannon’s entropy, and BDS values. The interaction terms between age and condition (HSP and ITW) reveal specific effects that appear when age is increased in impaired children. For HSP, this is reflected in the permutation entropy of pelvic and hip abduction and in the BDS value of flexion movements of the hip and the ankle. However, when walking speed increases in children suffering from HSP, differential changes in the pelvis are captured by Shannon’s entropy. In the case of ITW interacting with age, the combined effect considering permutation entropy, Shannon’s entropy, and BDS values is observed in many parameters of both proximal and distal joints; yet, an important reduction in the significance of the value of entropies appears when speed is introduced. Only the irreversibility values of pelvic flexion, knee rotation, hip flexion power, and mediolateral force are differentially changed.

### 3.3. Classification Scores per Group and Time-Series Type

**Figure 1:**
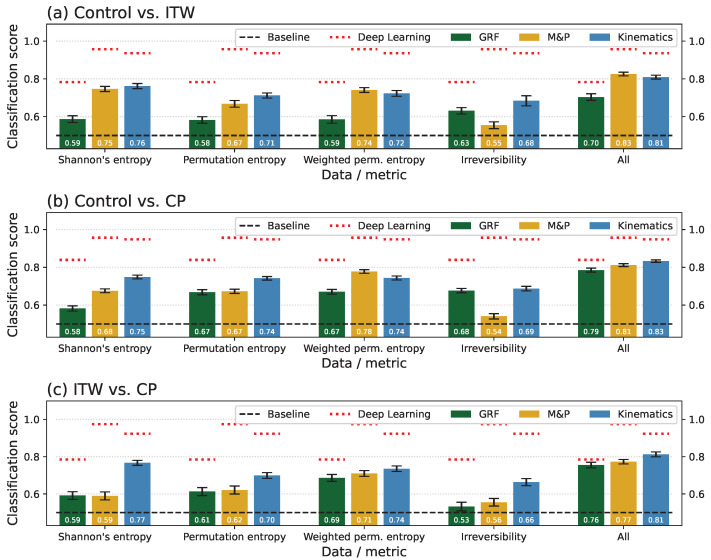
Synthesis of the classification scores obtained for the Dublin data set. The results are organised according to pairs of groups (horizontal panels), metrics (groups of bars), and types of time series (individual bars; see legends for colour codes). The horizontal red dotted lines report the scores obtained by the ResNet model on the same time series. The last group of bars, labelled “All”, corresponds to the classification using Random Forest and all statistical physics metrics together. The bars correspond to the average over 100 independent realisations and whiskers to the corresponding standard deviation. Finally, the horizontal dashed black lines indicate the baseline random classification score, i.e., 0.5.

We next assessed the results of statistical physics metrics by analysing how they allow for the differentiation of the groups of patients. For this, Figure 1 reports the classification scores for the Dublin data, organised by pairs of groups (see the three panels), metrics (groups of three bars; see bottom labels), and type of time series (GRF, green columns; M&P, ochre columns; and kinematics, blue columns). As additional references, the red dotted horizontal lines report the classification score obtained by the ResNet model on the same group of time series; and the final group of columns show the classification score obtained by applying a Random Forest model on all the statistical physics features.

Some initial insights can here be drawn. First of all, the statistical physics metrics yield classification scores above the 0.5 baseline, thus confirming that they capture relevant aspects of the gait dynamics that are different across groups. At the same time, individual metrics underperform when compared with the Deep Learning model but recover 80% of the score of the latter when combined together (see the last group of bars and also Figure A14 for a direct comparison). In short, individual metrics describe differences between groups focusing on specific aspects of the dynamics; only when they are combined together, they complement each other and yield a comprehensive picture. On the other hand, the fact that the Deep Learning model always achieves higher classification scores is not surprising, as ResNet represents the state of the art in time-series classification.

**Figure 2:**
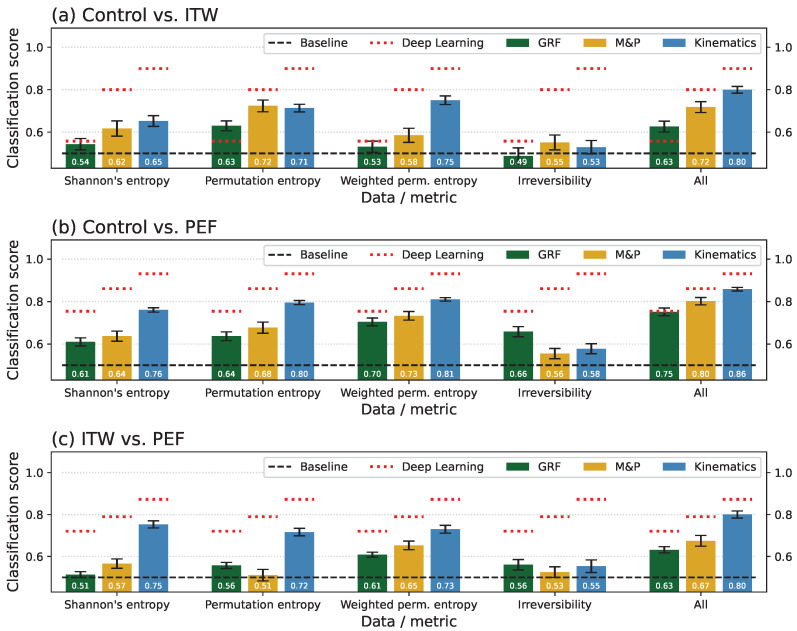
Synthesis of the classification scores obtained for the Madrilenian data set. The meanings of panels, columns, and colours are the same as in Figure 1.

When performing the same analysis on the Madrilenian data set, the results are qualitatively similar—see Figure 2 and Figure A13 for a comparison between statistical physics metrics and Deep Learning. A major difference can be found in the comparison between ITW and HSP patients, for which all metrics, except the weighted permutation entropy, seem to provide no useful information—the classification score is not statistically larger than 0.5.

### 3.4. Inter-Laboratory Comparison

Although a growing number of studies is contributing to describing gait pathologies, only a few publications clearly demonstrate the efficacy of 3D IGA as a decision-making tool [66]. The increase in inter-laboratory confidence will provide objective information and improved treatment planning, reinforcing consensus among clinicians and enhancing patient outcomes.

**Figure 3:**
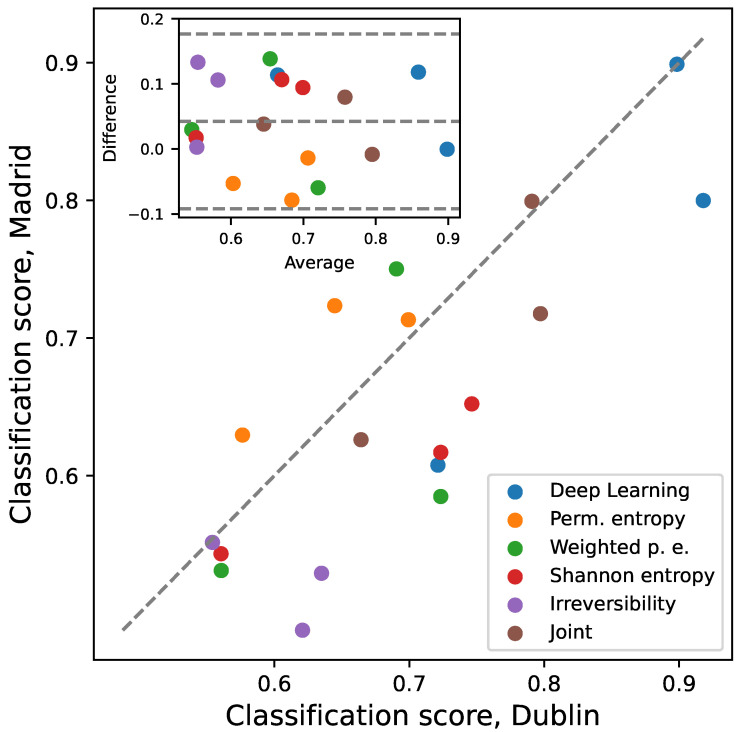
Classification scores, for different models and input features, obtained with the Madrilenian data as a function of the corresponding scores for the Dublin data. Points above (below) the main diagonal indicate that for the corresponding set of features, a better (respectively, worse) classification is obtained for the Madrilenian data than for the Dublin data. The classification task corresponds to control vs. ITW. See legend for colour codes. Inset: Bland–Altman plot of the same results.

As an initial assessment, Figure 3 reports a scatter plot of the classification scores obtained with the Madrilenian data, as a function of the corresponding score for the Dublin data. In order to make the comparison meaningful, the two groups present in both data sets (i.e., control subjects and ITW patients) are used in the classification. Different colours denote the input features used to train the RF and DL models. To illustrate, points above the main diagonal indicate that for the corresponding sets of features, a more precise classification is obtained for the Madrilenian data with respect to the Dublin data. Note that the latter data set includes a larger number of subjects; therefore, the models on it trained are expected to yield better precision. In order to eliminate such bias, the results for the Dublin data are recalculated considering 100 random subsets of subjects, of size equal to those available for the Madrilenian data. Importantly, the points are located near the main diagonal, with the latter being depicted by the grey dashed line. In other words, features seem to encode comparable quantities of useful information, consequently yielding similar classification scores in both data sets. This suggests that no systematic biases are present in the data—also confirmed by the Bland–Altman plot in the inset.

**Figure 4:**
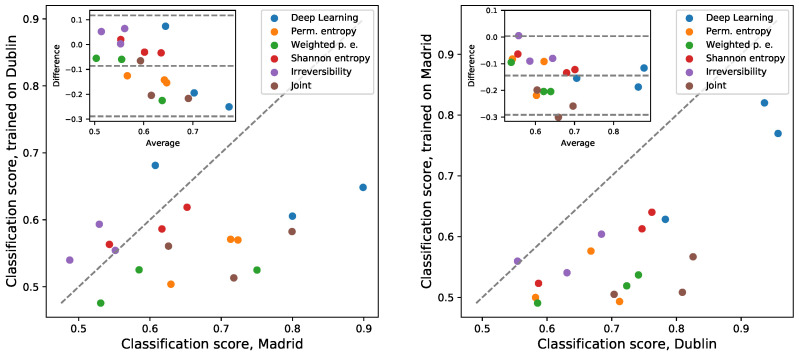
Model cross-applicability. The left panel reports the classification scores obtained when the models are trained and evaluated on the Dublin and Madrilenian data as a function of the same scores obtained only using Madrilenian data. The right panel reports the results for the inverted scenario. The points below the main diagonal indicate that information is lost when a model is trained on one data set and then applied to the other data set. Data sets, colour codes, and insets as per Figure 3.

As a further analysis, Figure 4 reports the results obtained with a cross-application of the models. The left panel reports the score obtained for a given model and input features, when such model is trained over the Dublin data and evaluated on the Madrilenian data (Y-axis); and such score is compared to the one obtained when both training and validation are performed on the Madrilenian data set (X-axis). The right panel of the same figure corresponds to the inverted scenario. These two panels thus allow us to visualise what happens when the models are trained and validated on the data of different laboratories; the points below the main diagonal indicate that the two data sets are not compatible. It can be appreciated that now, most results are significantly below the main diagonal; hence, the data recorded in the two locations are different from the point of view of the DL model.

In synthesis it can be concluded that both data sets contain a similar quantity of relevant information, as the condition can be classified in both cases with similar precision (Figure 3). Yet, such information is not the same, and the resulting models are not equivalent (Figure 4). From a practical point of view, in spite of using similar equipment and protocols, the models developed in one laboratory cannot be used on the data recorded in another one.

### 3.5. Analysis of Feature Importance in Statistical Physics Models

The classification results from the Random Forest model are further analysed based on the relative importance of the features used to discriminate between groups. To show their effect on the model, the variable with the highest improvement score is set as the most important, and the rest of them follow in order of relevance. Numerically, the most significant feature has always a relative importance value of 1.0, which gradually decreases for the rest of parameters. Figure A8, Figure A9 and Figure A10 show the most relevant variables used by the Random Forest model to differentiate between groups in the Dublin data. From the GRF section, discrepancies can be observed in the comparisons with CP (Figure A9 and Figure A10), where the most important variable is the irreversibility in the sagittal component, and with ITW (Figure A8), where the relevance is similarly shared between different entropies and planes. In moments and powers (M&P), the most significant variable for all classifications is the irreversibility measured for the knee power, followed distantly by other entropies for the hip and the knee. On the other hand, entropies appear to have the highest relative importance in kinematics. In ITW, when compared against the control (Figure A8) and CP (Figure A10), the weighted entropy of the knee in the frontal plane stands out from the rest. However, in CP vs. control (Figure A9), the most relevant feature is the weighted entropy of pelvic abduction–adduction, followed closely by the Shannon’s entropy of the hip in the sagittal plane. This may partly be explained by the fact that the objective of gait is the realisation of a forward linear movement; in the presence of gait alterations, other planes may require larger compensatory changes in order to achieve the same movement, which stand out in an instrumental analysis.

For the Madrilenian lab, in Figure A11, Figure A12 and Figure A13, we can observe a detailed landscape of the relevant variables. Entropies from the sagittal plane are predominant in the GRF, although irreversibilities from the sagittal and anteroposterior components in HSP vs. control and ITW vs. control, respectively, show relative importance. As with the case of Dublin data, the most important features of M&P in ITW compared with the healthy controls (Figure A11) are irreversibilities, specifically of the hip and ankle moments. However, this contrasts with the relevant irreversibilities of the knee and hip powers observed in the Dublin comparison. For HSP patients, the most relevant parameters are the permutation entropies of the knee power when compared with the controls and of the ankle power when compared with ITW. In kinematics, there also differences in the Dublin ITW vs. control comparison. Instead of the entropy of the knee, the most relevant variable now is the permutation entropy of the hip in the transversal plane, with other important features being the irreversibility of ankle dorsiflexion or the Shannon’s entropy of the pelvis. In the comparison with HSP patients, the significant parameters are found predominantly in the transversal plane, as is the case of the weighted permutation entropy of the ankle when compared with ITW and the knee irreversibility when compared with the controls.

In general, we can observe that entropies are comparably more important for the GRF and kinematics classification, while irreversibilities dominate in M&P. Furthermore, for the Dublin data, there is often a greater difference in importance between the most relevant variable and the next, while for the Madrilenian data, a similar relative importance is shared between many features. It is relevant to say that while a feature might hold the highest relative importance in the model, when applied in conjunction with other predictors, the classification score might reduce due to, for example, interaction effects. This can be seen in some relevant individual irreversibilities in M&P that achieve the lowest classification score in the general model (Figure 1 and Figure 2).

### 3.6. Classifications Based on Individual Time Series

In order to better understand the behaviour of Deep Learning models, we trained them using individual time series.

**Figure A1:**
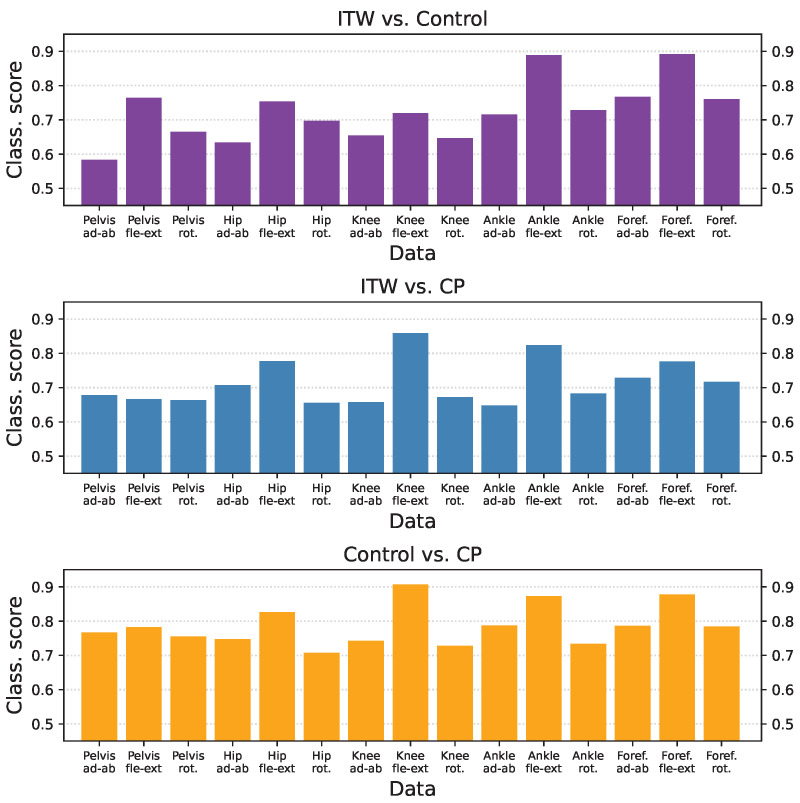
Deep Learning classification scores for Dublin data, using single kinematic time series. Horizontal panels represent comparison between pairs of groups, and each coloured bar is an individual time series. *Foref.*: forefoot; *ad-ab*: adduction–abduction; *fle-ext*: flexion–extension; *rot.*: rotation.

In the Dublin kinematic data (Figure A1), the best classification scores are given by ankle and foot flexion models when comparing ITW vs. control, knee and ankle flexion models when classifying ITW vs. CP, and knee, ankle, and foot flexion models when assessing ITW vs. control. In the Madrid kinematic data (Figure A3), they were given by ankle and foot flexion models when comparing ITW vs. control, knee and ankle when classifying HSP. vs. control, and foot flexion models when assessing ITW vs. HSP.

**Figure A2:**
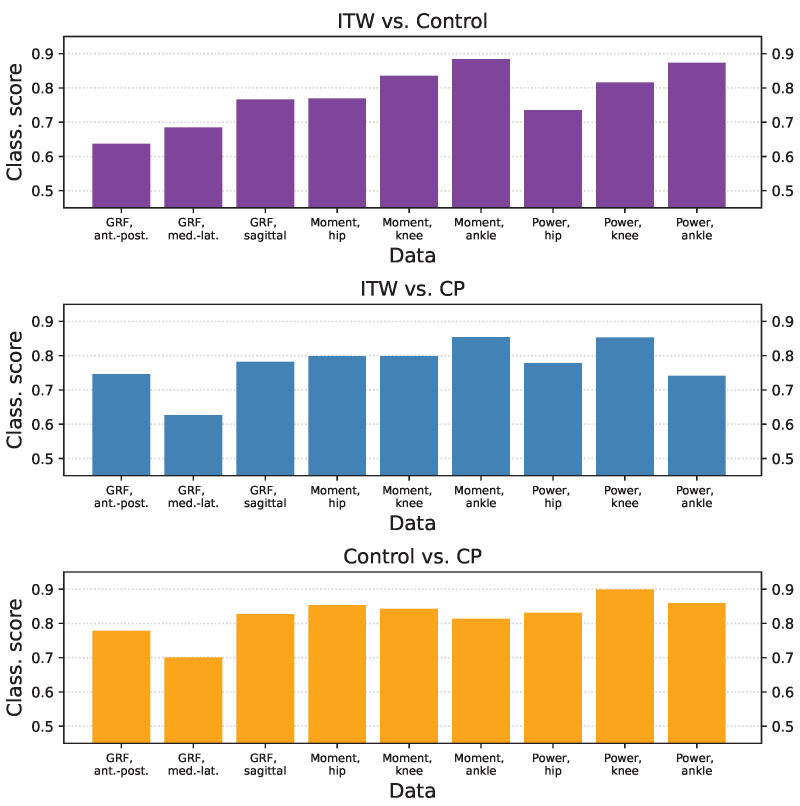
Deep Learning classification scores for Dublin data, using single GRF and M&P time series. Meanings of panels and coloured bars as per Figure A1. *Ant.-post*: anteroposterior; *med.-lat.*: mediolateral.

**Figure A3:**
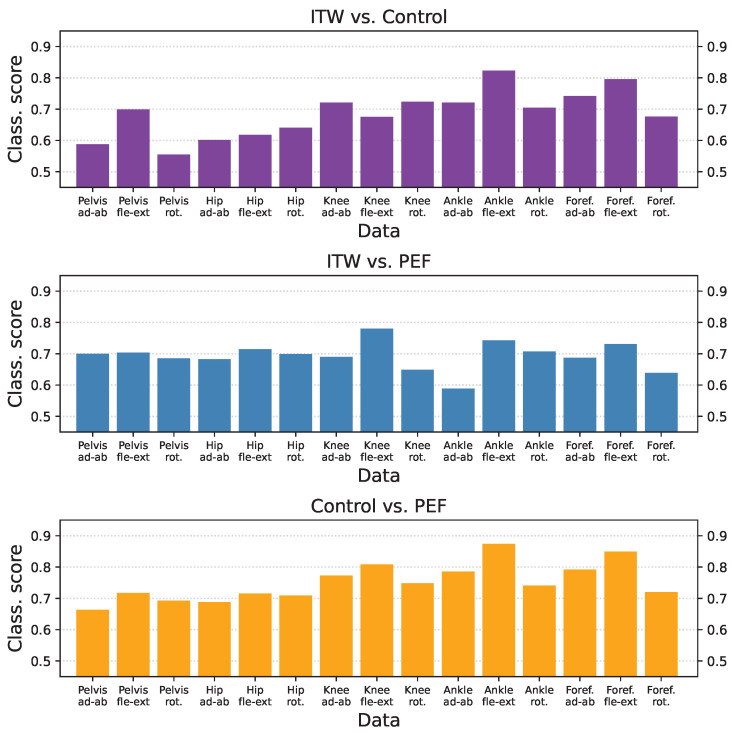
Deep Learning classification scores for Madrilenian data, using single kinematic time series. Meanings of panels, coloured bars, and labels as per Figure A1.

**Figure A4:**
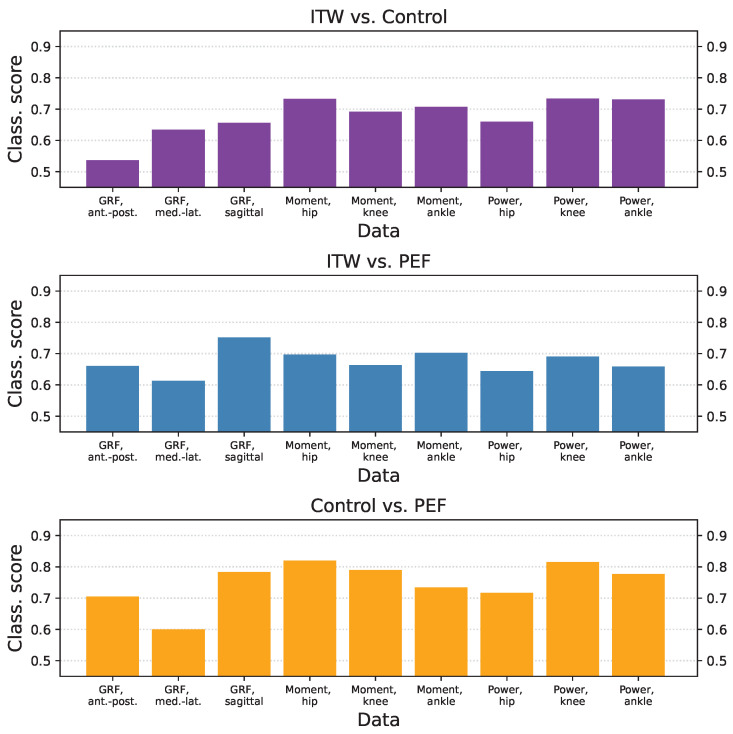
Deep Learning classification scores for Madrilenian data, using single GRF and M&P time series. Meanings of panels, coloured bars, and labels as per Figure A2.

**Figure A5:**
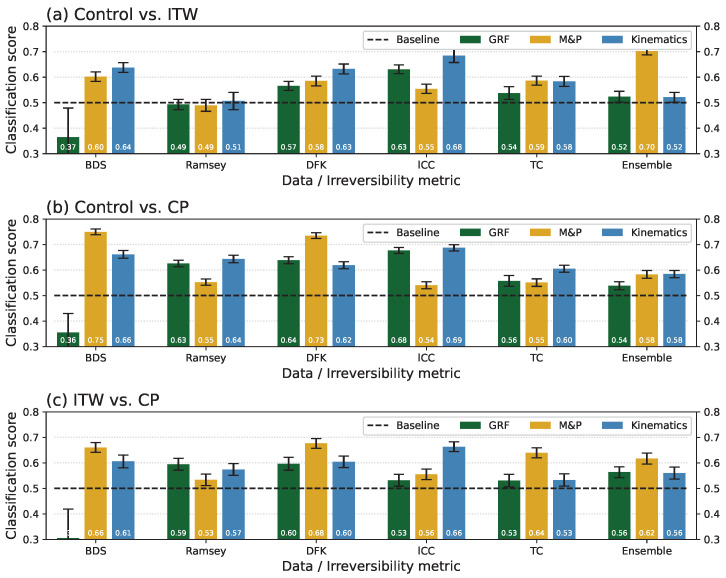
Classification scores obtained by irreversibility metrics in Dublin data using Random Forest model. Results are organised according to pairs of groups (horizontal panels), irreversibility metrics (groups of bars), and types of time series (individual bars; see legends for colour codes). Bars correspond to averages over 100 independent realisations and whiskers to the corresponding standard deviations. Finally, horizontal dashed black lines indicate baseline random classification score, i.e., 0.5.

**Figure A8:**
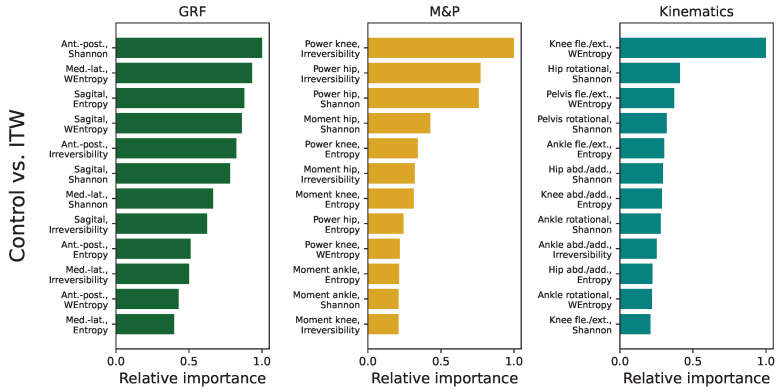
The ranking of the most important features in control vs. ITW for the Dublin data. Each vertical panel represents a type of time series, and each horizontal bar represents the relative importance of the individual metric. The relative importance interval ranges from 0 to 1, with the most important variable always being 1 and the rest gradually decreasing according to the classification relevance. *Ant.-post*: anteroposterior; *med.-lat.*: mediolateral; *add./abd.*: adduction–abduction; *fle./ext.*: flexion–extension.

**Figure A9:**
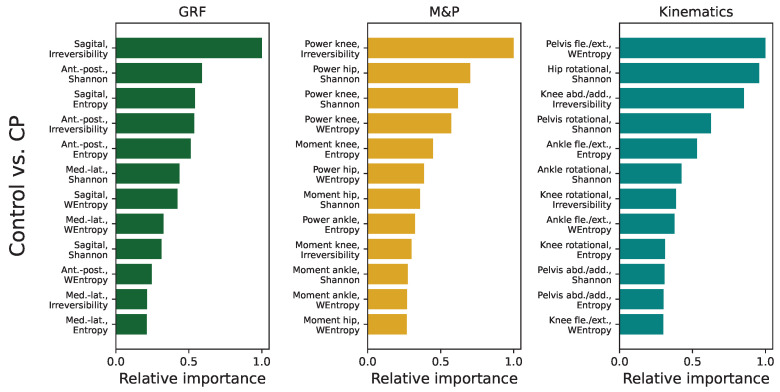
The ranking of the most important features in control vs. CP for the Dublin data. Meanings of panels and labels as per Figure A8.

**Figure A10:**
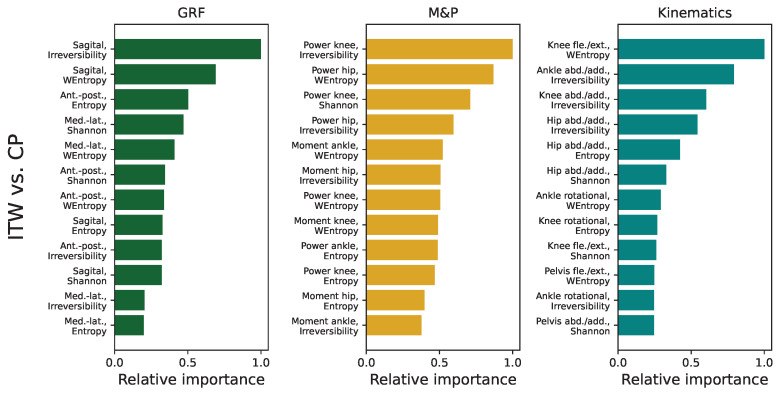
The ranking of the most important features in ITW vs. CP for the Dublin data. Meanings of panels and labels as per Figure A8.

**Figure A11:**
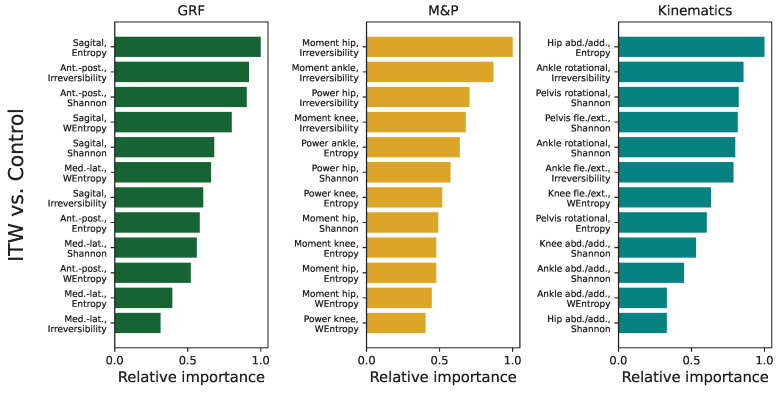
The ranking of the most important features in control vs. ITW for the Madrilenian data. Meanings of panels and labels as per Figure A8.

**Figure A12:**
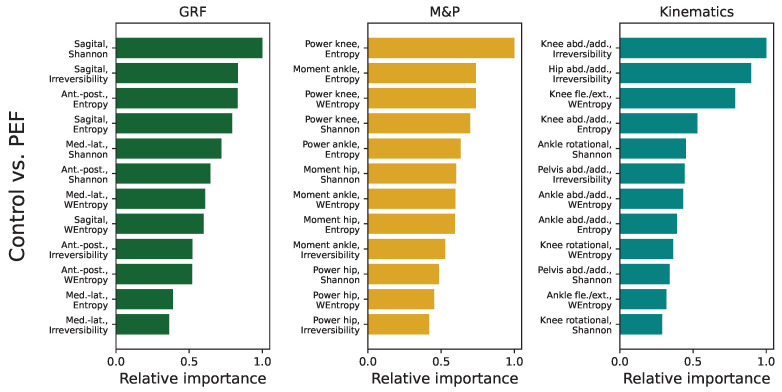
The ranking of the most important features in control vs. HSP, for the Madrilenian data. Meanings of panels and labels as per Figure A8.

**Figure A13:**
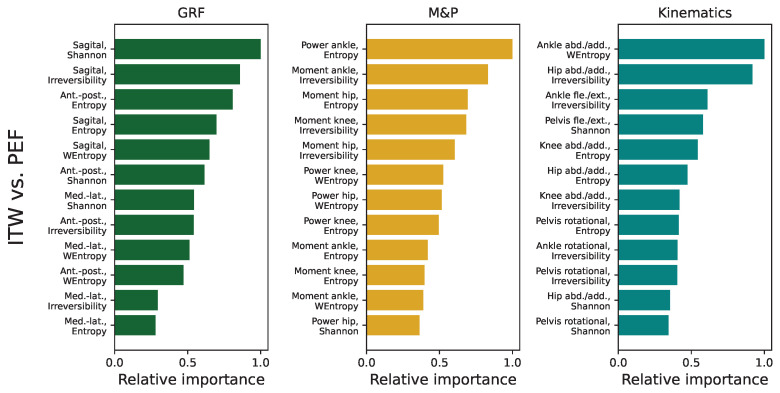
The ranking of the most important features in ITW vs. HSP, for the Madrilenian data. Meanings of panels and labels as per Figure A8.

The classification scores for the sagittal GRF were better than the anteroposterior and mediolateral GRF time series for all the classification scenarios. Models using moment and power time series tended to have similar performances. In the Dublin data, ankle moment and power were the best models for ITW vs. control; knee moment, knee and ankle power, and hip moment for ITW vs. CP; and ankle moment and power for ITW vs. CP. In the Madrilenian data, ITW vs. control were better classified when the models were trained with the hip moment, and knee and ankle power time series. Hip and ankle moment models were the best for HSP vs. ITW, and hip and knee moment and knee power for HSP vs. control. See Figure A2 and Figure A4.

### 3.7. Optimal Resolution of Time Series

While gait is a continuous movement and can thus, in theory, be described by continuous waveforms, a digital data analysis necessarily requires such curves to be discretised; consequently, one problem is the identification of the best time resolution to describe gait. This comes as a balance between two extremes. On one hand, it is intuitive that using too-coarse-grained time series can result in losing relevant data. On the other hand, sampling at higher-than-necessary frequencies can result in unnecessarily large computational costs but also in the inclusion of noisy information that could be detrimental for the analysis.

A single gait cycle has been represented in the literature as time series of varied length, from as low as 11 points [67] to over 1500 [68], with most of the research works agreeing on an intermediate value of 100 points—i.e., to the percentage occurrence per step cycle; see [69,70,71,72,73].

We here analyse this issue by evaluating the score obtained by the DL model as a function of the length of the time series representing one single gait cycle; see Figure A16. In agreement with what previously reported [67], increasing the resolution above 50 points per cycle yields no added value—except for a few cases, e.g., the Y-axis of the hip in the ITW vs. CP classification, where the improvement is, in any case, minimal. The optimum seems to be always located between 20 and 30 points per cycle. Finally, considering time series of more than 100 values leads to a minor yet noticeable drop in the classification score.

### 3.8. Analysis of Gait Sub-Windows

We finally analyse the impact of selecting particular intervals of the gait cycle on the classification score. In Figure A17, we show the classification scores for different initial points and lengths. In general, we can say that the model performances do not decrease in a dramatic way when selecting a shorter windows of data, with the exception of the short sub-windows of the GRF corresponding to data only from the swing phase (starting point at 60% or higher).

Classifications based on the GRF are the ones that are more altered by the length and starting point of the sub-windows. The best sub-windows for the GRF are those starting at around 0–20% of the cycle with a length of around 40–80% of the gait cycle. The classification scores of the sub-windows based on moments and powers are better than those for the GRF data, and they generally show large classification scores. The best sub-windows based on moments and powers differ depending on the classification problem. In control vs. ITW, the best classification scores are those focused on the first parts of the gait cycle, starting at around 0–20% of the cycle with a length of around 40–80% of the gait cycle. In control vs. CP, the best sub-windows are those that focus on the second half of the gait cycle (starting at around 20 to 40%, with lengths of 40 to 80%). In ITW vs. CP, the best sub-windows start at around 20–40% with lengths of around 40–80%, meaning that the model focuses on the second and third parts of the gait cycle. Although the global classification performance is also high for the kinematic time series, there are also optimal sub-windows. In control vs. ITW, the best sub-windows are those starting at around 0–20% with lengths of 40–80% of the gait cycle, meaning that segments of the initial contact and early stance of the cycle are the most critical to classification. In control vs. CP, the best sub-windows start at around 20–60% with lengths of 40–80%, meaning that the critical segments of the gait cycle for classification are those from the second half of the gait cycle (equivalent to M&P). In ITW vs. CP, the optimal sub-windows start at around 20–40% with lengths of 40–80% of the gait cycle, focusing on the second and third parts of the gait cycle, as in M&P.

## 4. Discussion

The results presented throughout this work can be analysed from three different viewpoints: one focused on data analysis, a second one focused on neuromotor control, and the last dealing with medical implications.

### 4.1. Discussion from the Data Analysis Perspective

The first technical consideration regards data inter-operability. This does not only refer to the fact that data coming from different laboratories must have a similar format from an IT point of view [74]; they must also have been recorded under similar conditions, in order for a classification or diagnostic model developed in one laboratory to be usable in another location. In spite of having been recorded with the same equipment and following very similar protocols, the two data sets here considered are not equivalent, and a model trained on one of them does not yield comparable results when applied to the other one—see Figure 3 and Figure 4. Several factors may be behind this, for instance, variations in equipment calibration, the environmental conditions under which the gait analysis is conducted, or differences in patients’ characteristics. Two solutions can be envisioned to tackle this issue. First of all, one could isolate all variables making the data sets different and design a standardised and unified protocol—something that would require a collaborative large-scale study. Secondly, a simpler solution may involve publishing models trained over data combining recordings performed in different laboratories, in order to obtain an “average model” including only information common in all sources. This is possible thanks to the sensitivity of DL models. To illustrate, a ResNet model trained to discriminate control subjects and ITW patients with mixed data from both laboratories still yields excellent results: 0.75 ± 0.03 for the GRF, 0.92 ± 0.03 for M&P, and 0.92 ± 0.02 for kinematics (average ± standard deviation over 100 independent realisations).

As a second technical consideration, we here studied the effect of time-series resolution in the classification score and hence in the quantity of information actually encoded in the time series. The optimal value seems to be between 20 and 30 measurements per walking cycle (see Figure A16), i.e., on the low side of what usually considered in the literature [67]. Additionally, in most cases, the full gait cycle is not needed, and good results can be obtained by only considering its middle part (see Figure A17), which could reflect the importance of the weight transfer mechanism to differentiate between conditions. It is important to note that these results are problem-dependent. In other words, while high-resolution time series are not needed when these are classified by Deep Learning models, this does not imply that a lower resolution will always be a better option. Still, the fact that low-resolution time series are enough for the problem at hand is clearly beneficial from a computational cost viewpoint; and it could also open the door to the use of less expensive and more flexible recording options, e.g., the analysis of videos [75,76,77].

Another relevant issue is the availability of different biomechanical models that could be chosen for kinematic and kinetic data evaluation. In our case, we selected the commercial solution provided by the manufacturer, i.e., the modified Helen Hayes model, to increase inter-laboratory operability with the same laboratory equipment. However, this could also imply some challenges in the out-of-sagittal plane [78] and may limit the calculation of related kinetic data.

**Figure A14:**
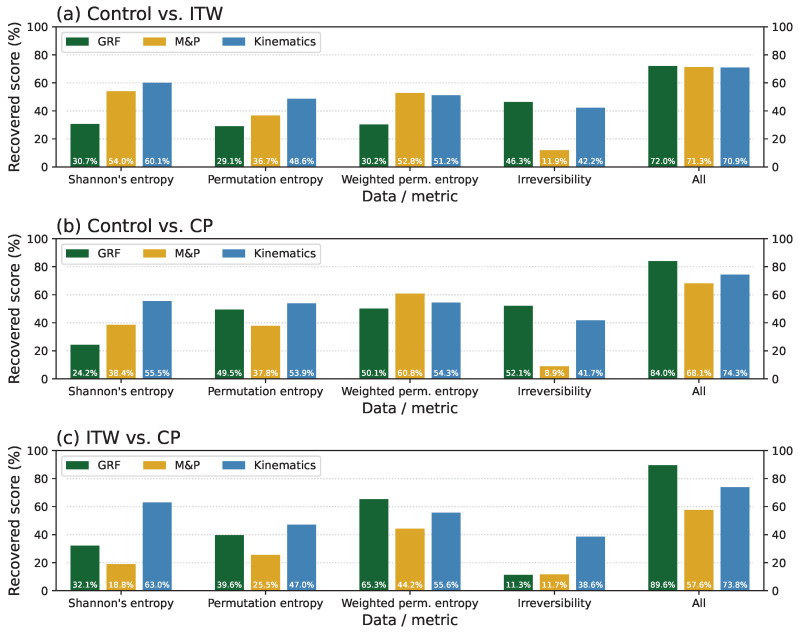
Percentages of the scores recovered by each Random Forest model when compared with the score obtained by the Deep Learning classification for the Dublin data set. Meanings of panels, groups of bars, and colours as per Figure 1.

**Figure A15:**
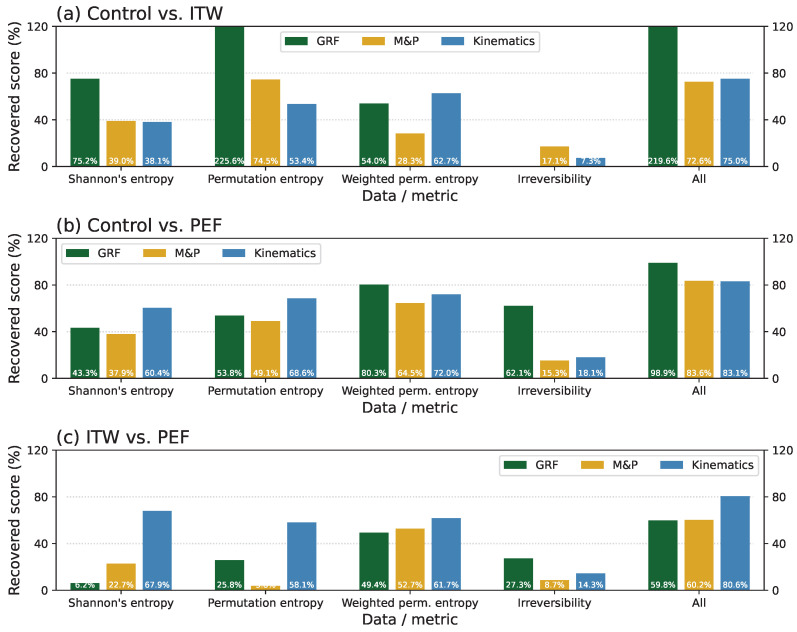
Percentages of the scores recovered by each Random Forest model when compared with the score obtained by the Deep Learning classification for the Madrilenian data set. Meanings of panels, groups of bars, and colours as per Figure 2.

**Figure A16:**
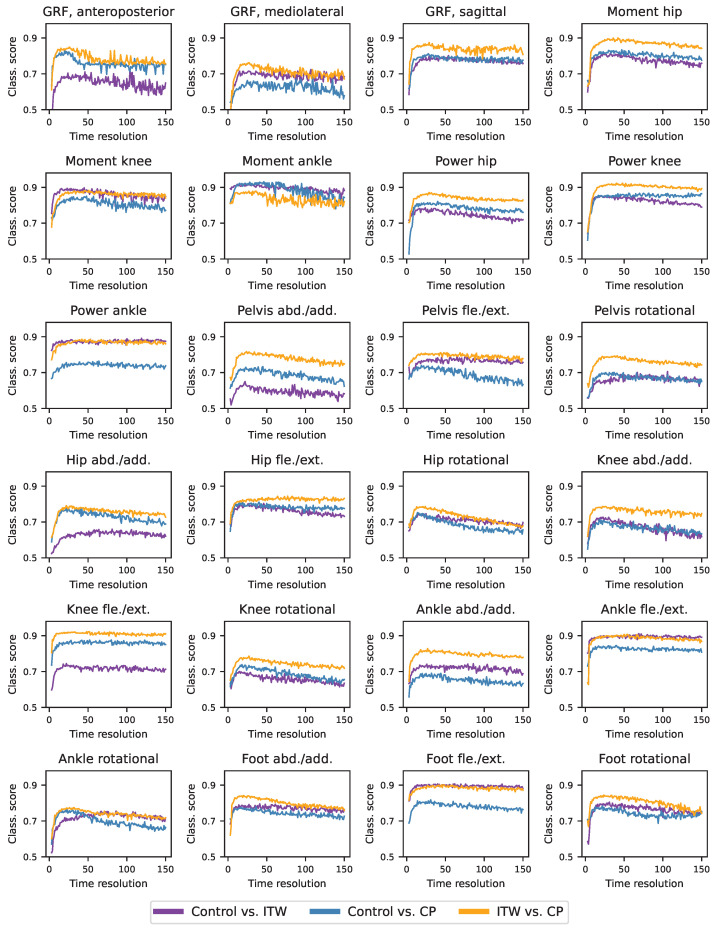
Evolution of the classification scores obtained by the DL model as a function of the length (and hence of the temporal resolution) of the time series. Each panel represents an individual time-series type (see title), and the three lines in each of them refer to the classifications of control subjects vs. ITW (purple line), control subjects vs. CP (blue line), and ITW vs. CP (orange line). *Abd./add.*: abduction–adduction; *fle./ext.*: flexion–extension.

**Figure A17:**
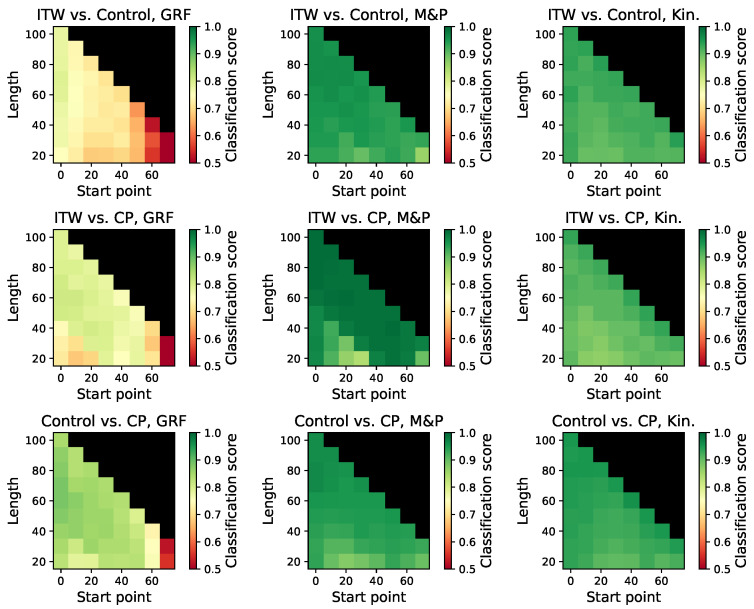
Analysis of gait sub-windows. Each panel reports the classification scores obtained considering sub-windows of the original time series, as a function of the starting point (X-axes) and length (Y-axes). The initial time series has a length of 100 points in all cases. The results correspond to the average scores obtained by 100 independent ResNet Deep Learning models. The classification task and type of data used are reported in the title of each panel.

**Figure A18:**
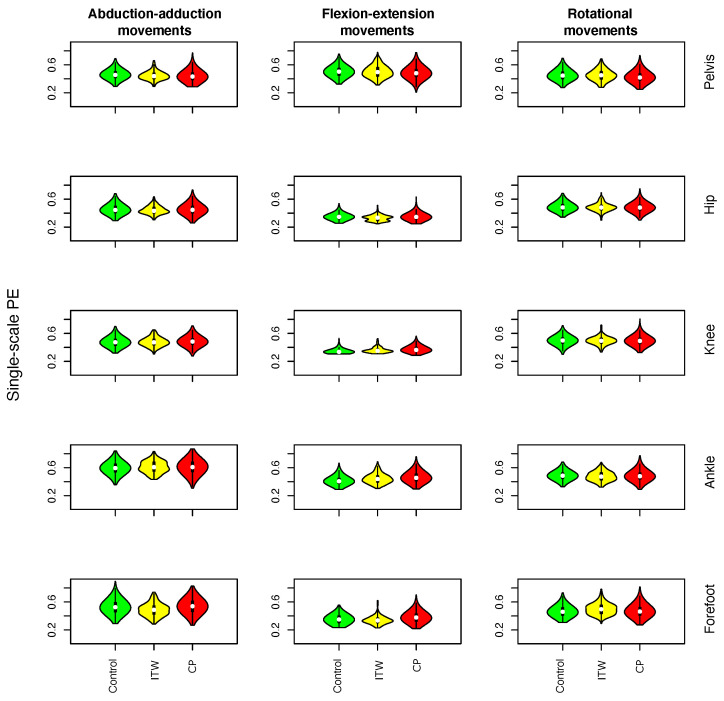
Probability distributions of the permutation entropy (PE) from kinematic time series, as calculated in control subjects and patients with idiopathic toe walking (ITW) and cerebral palsy (CP) from the Dublin lab. Rows, from top to bottom, correspond to pelvis, hip, knee, ankle, and forefoot joints, respectively, and columns, from left to right, to the abduction–adduction, flexion–extension, and rotational axes.

**Figure A19:**
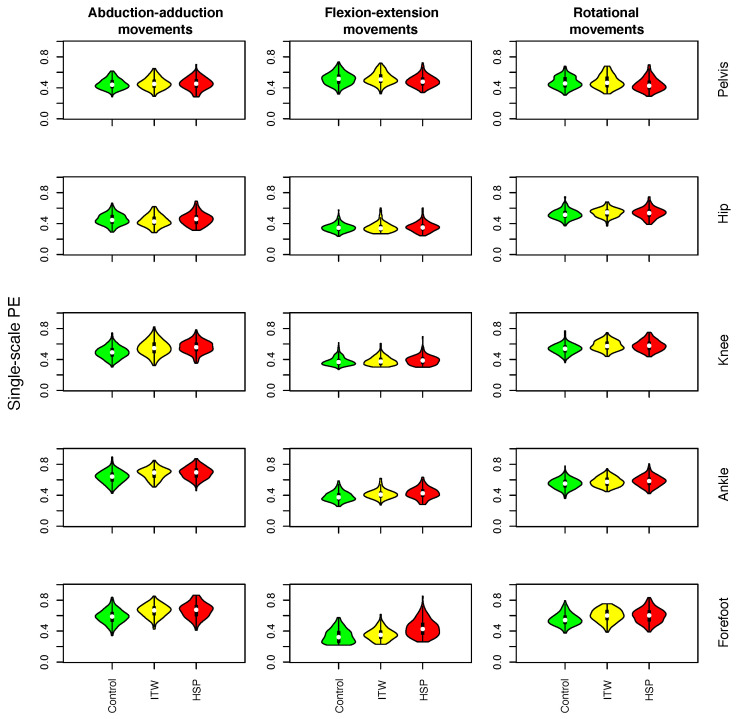
Probability distributions of the permutation entropy (PE) from kinematic time series, as calculated in control subjects and patients with idiopathic toe walking (ITW) and hereditary spastic paraplegia (HSP) from the Madrilenian lab. Meanings of rows and columns as per Figure A18.

**Figure A20:**
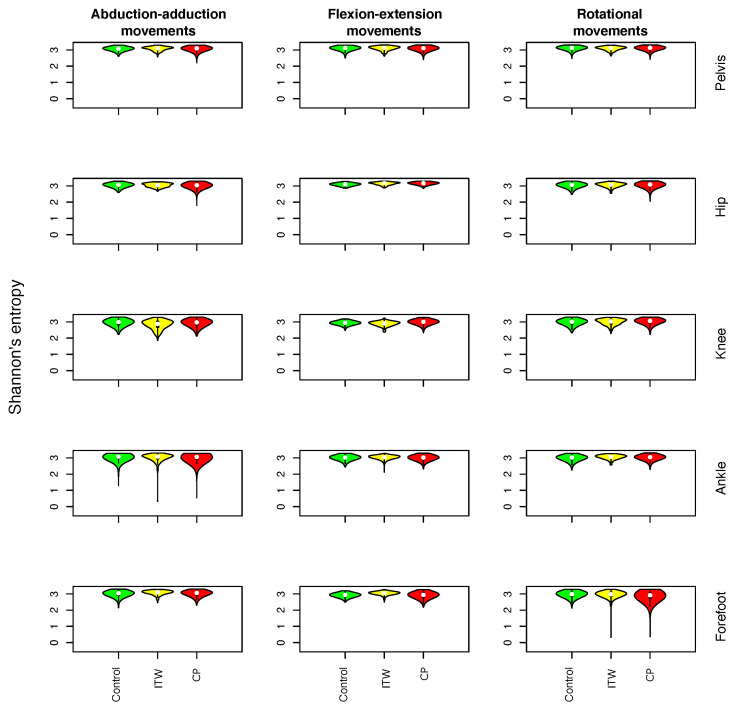
Probability distributions of Shannon’s entropy (SE) from kinematic time series, as calculated in control subjects and patients with idiopathic toe walking (ITW) and cerebral palsy (CP) from the Dublin lab. Meanings of rows and columns as per Figure A18.

**Figure A21:**
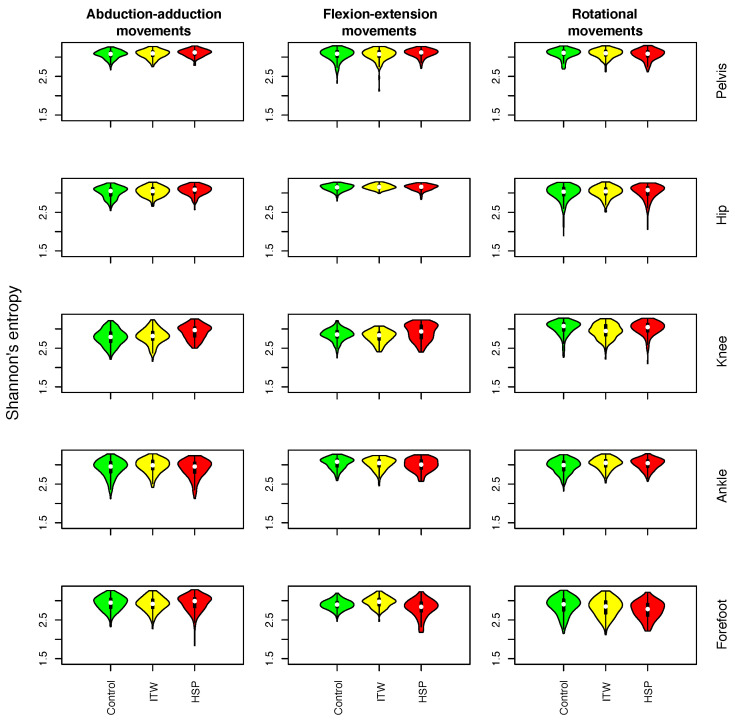
Probability distributions of Shannon’s entropy (SE) from kinematic time series, as calculated in control subjects and patients with idiopathic toe walking (ITW) and hereditary spastic paraplegia (HSP) from the Madrilenian lab. Meanings of rows and columns as per Figure A18.

**Figure A22:**
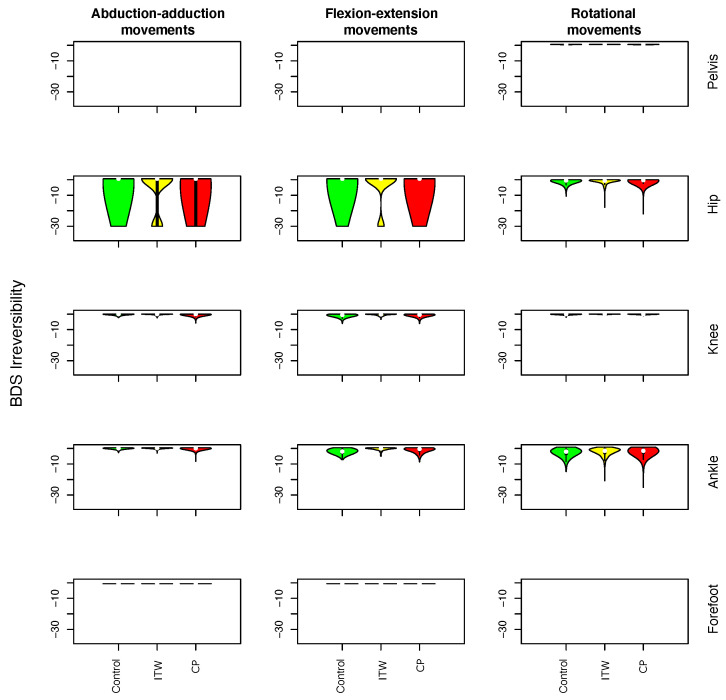
Probability distributions of BDS irreversibility from kinematic time series, as calculated in control subjects and patients with idiopathic toe walking (ITW) and cerebral palsy (CP) from the Dublin lab. Meanings of rows and columns as per Figure A18.

**Figure A23:**
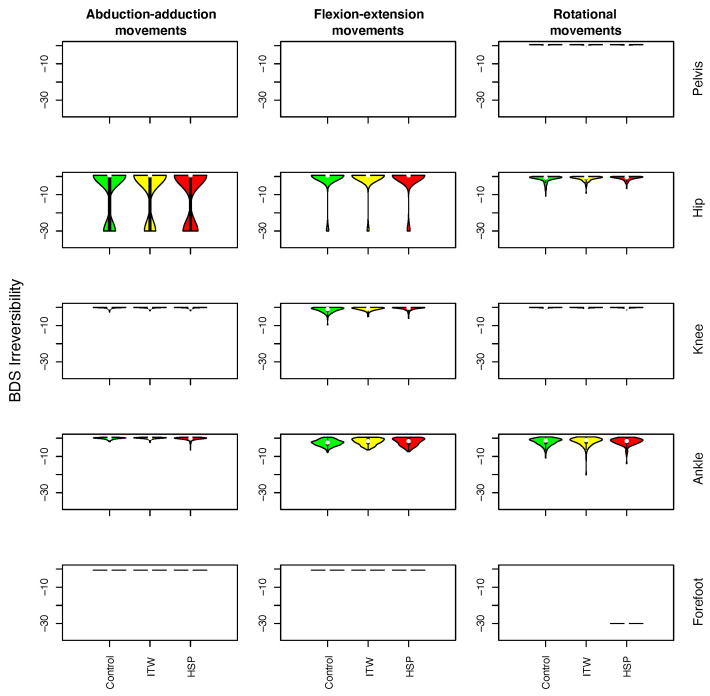
Probability distributions of BDS irreversibility from kinematic time series, as calculated in control subjects and patients with idiopathic toe walking (ITW) and hereditary spastic paraplegia (HSP) from the Madrilenian lab. Meanings of rows and columns as per Figure A18.

**Figure A24:**
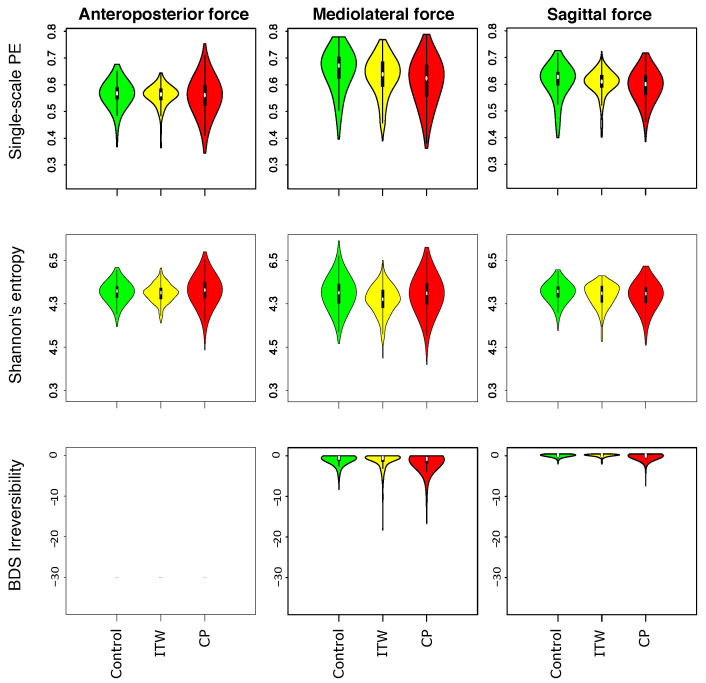
Probability distributions of complexity metrics from ground reaction force (GRF) time series, as calculated in control subjects and patients with ITW and CP from the Dublin lab. Rows, from top to bottom, correspond to permutation entropy (PE), Shannon’s entropy (SE), and BDS irreversibility, respectively, and columns, from left to right, to the sheer anteroposterior, sheer mediolateral, and sagittal forces.

**Figure A25:**
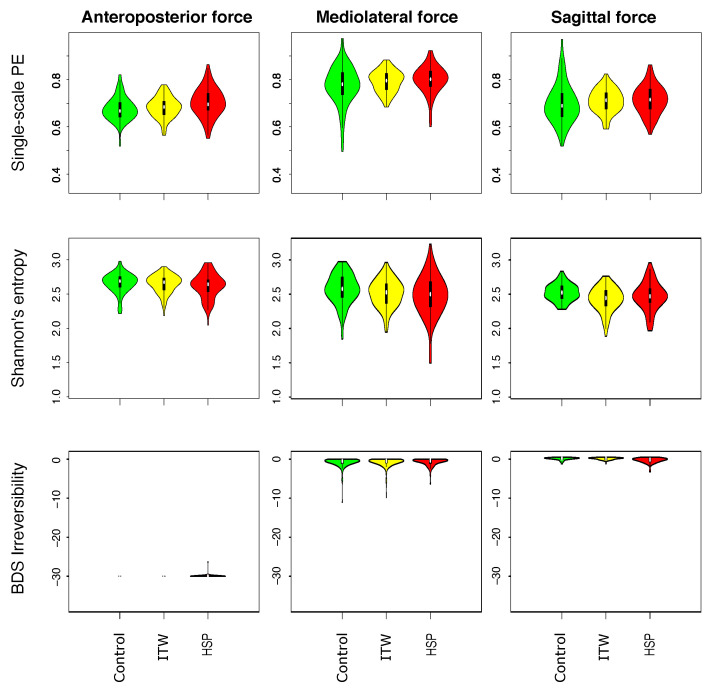
Probability distributions of complexity metrics from ground reaction force (GRF) time series, as calculated in control subjects and patients with ITW and HSP from the Madrilenian lab. Meanings of rows and columns as per Figure A24.

**Figure A26:**
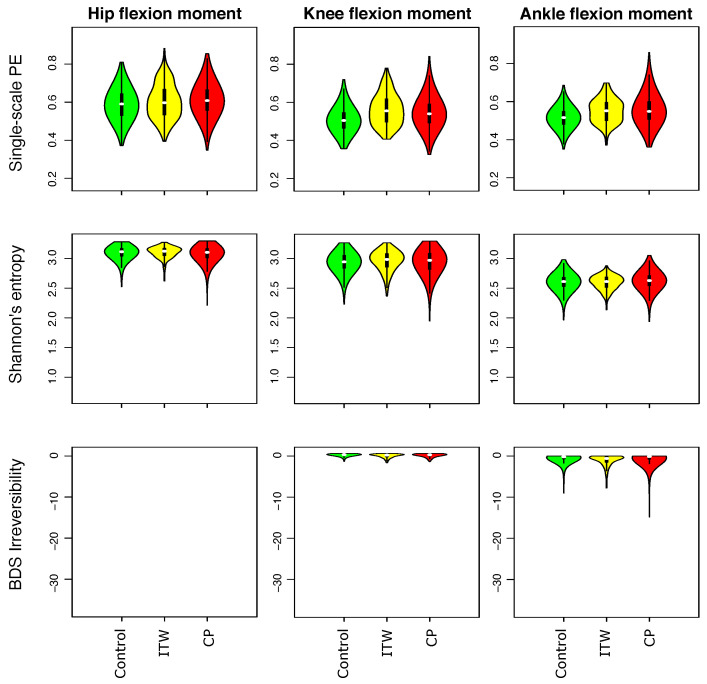
Probability distributions of complexity metrics from joint moment time series, as calculated in control subjects and patients with ITW and CP from the Dublin lab. Rows, from top to bottom, correspond to permutation entropy (PE), Shannon’s entropy (SE), and BDS irreversibility, respectively, and columns, from left to right, to hip, knee, and ankle flexion moments.

**Figure A27:**
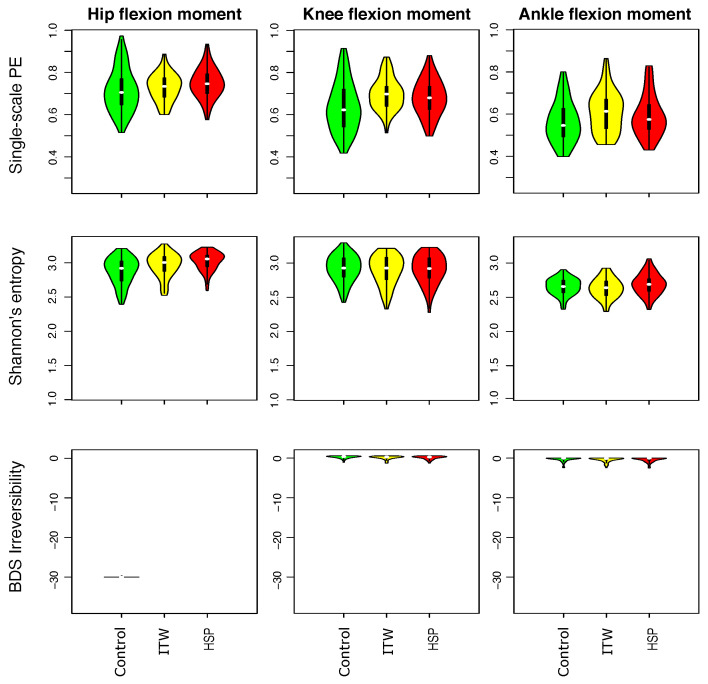
Probability distributions of complexity metrics from joint moment time series, as calculated in control subjects and patients with ITW and HSP from the Madrilenian lab. Meanings of rows and columns as per Figure A26.

**Figure A28:**
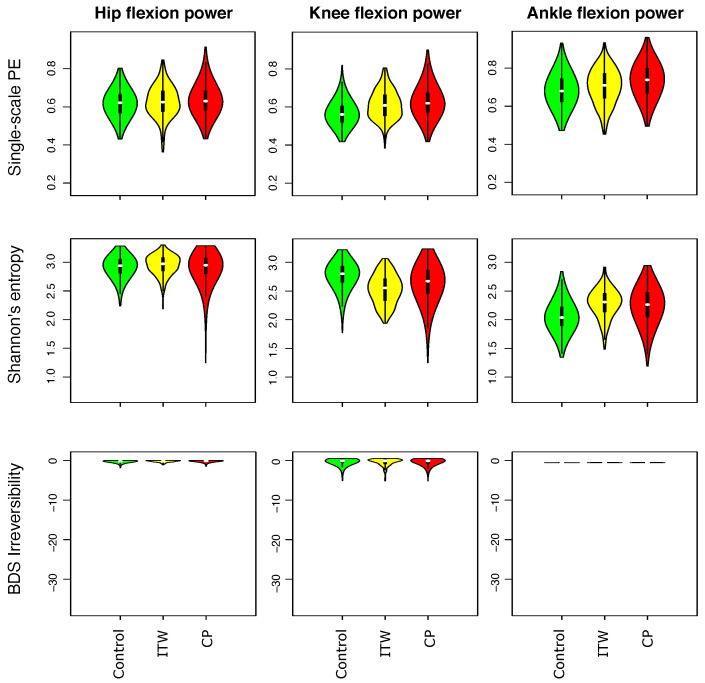
Probability distributions of complexity metrics from joint power time series, as calculated in control subjects and patients with ITW and CP from the Dublin lab. Rows, from top to bottom, correspond to permutation entropy (PE), Shannon’s entropy (SE), and BDS irreversibility, respectively, and columns, from left to right, to hip, knee, and ankle flexion powers.

**Figure A29:**
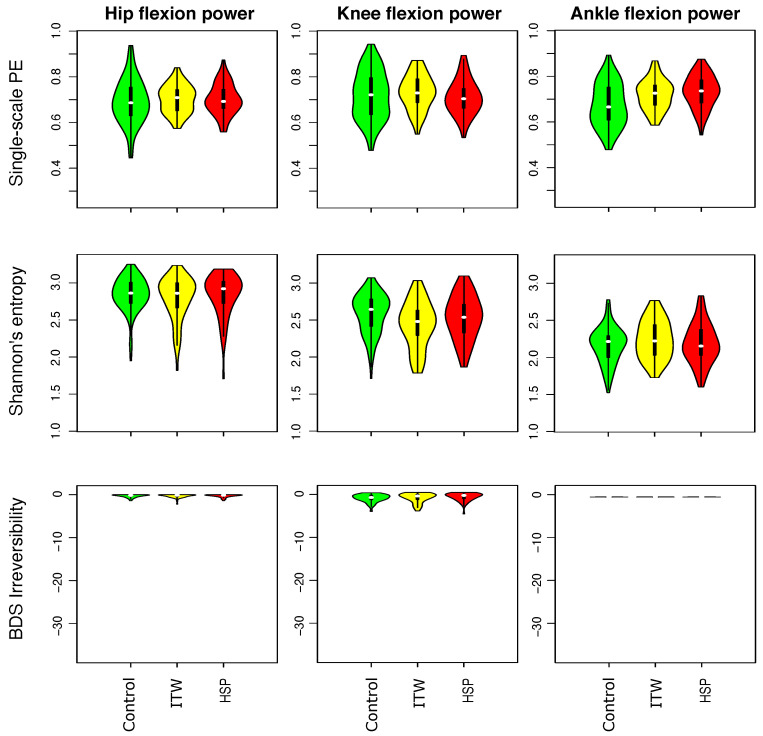
Probability distributions of complexity metrics from joint power time series, as calculated in control subjects and patients with ITW and HSP from the Madrilenian lab. Meanings of rows and columns as per Figure A28.

Finally, the results here presented allow us to evaluate the use of statistical physics metrics for the study of gait disorders, a topic that has hitherto received moderate attention from the scientific community [33]. These metrics provide a completely different approach to the study of gait. On one hand, they focus on specific properties of the time series based on a priori principles of dynamical systems and thus not on gait-specific characteristics—i.e., they are not tailored to the problem here tackled. On the other hand, they are inherently over-simplifications: a whole time series is synthesised in one single value. In spite of this, they achieve notable results when combined, recovering up to 80% of the classification score of Deep Learning models—see Figure A14 and Figure A15. This suggests that they are able to capture essential differences between groups of subjects. At the same time, metrics underperform when considered individually (see Figure 1 and Figure 2), indicating that they yield complementary views on the underlying dynamics. While entropy and irreversibility metrics have been considered before in gait analysis [26,27,28,31,32,33], their complementary nature has received less attention.

### 4.2. Insights into Motor Control Through Statistical Physic Metrics and Deep Learning

We have shown how Shannon’s entropy, permutation entropy, and BDS irreversibility of joint kinematic and kinetic time series are modified in different gait conditions—for the sake of clarity, detailed results are presented in Figure A18, Figure A19, Figure A20, Figure A21, Figure A22, Figure A23, Figure A24, Figure A25, Figure A26, Figure A27, Figure A28 and Figure A29.

The use of entropy metrics allows for the assessment of the complexity or predictability of a system, in terms of the amplitude of a time series and its dynamic or temporal organisation, while the time irreversibility holds information about its memory. In biological terms, this is translated into metrics that may help us understand the differences in motor control and neuromuscular adaptations in children.

We have demonstrated that healthy children show different patterns of statistical physic metrics. Permutation and Shannon’s entropy values are lower in multiple joint gait series in healthy children, indicating a more predictable and uniform gait. The BDS irreversibility metrics in these children also show consistent trends, suggesting a balanced and regular gait pattern without the irregularities that may appear in gait abnormalities.

**Figure A30:**
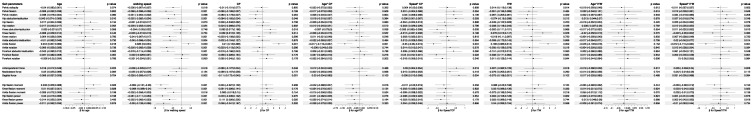
Forest plots showing the effect of age, normalised walking speed, cerebral palsy (CP), idiopathic toe walking (ITW), and the interaction effects between each condition and both age and normalised walking speed on the permutation entropy (PE) of the kinematic and kinetic gait time series for CP, ITW, and healthy children. Each row represents an individual time series or gait parameter. The black square indicates the value of the regression coefficient “*β*” for each variable of the linear mixed model. The horizontal line represents the 95% confidence interval estimated by the bootstrap; if the line does not cross the zero line (or large vertical line), it is a reliable estimation.

**Figure A31:**
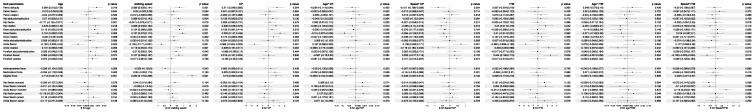
Forest plots showing the effect of age, normalised walking speed, cerebral palsy (CP), idiopathic toe walking (ITW), and the interaction effects between each condition and both age and normalised walking speed on the Shannon’s entropy (SE) of the kinematic and kinetic gait time series for CP, ITW, and healthy children. Each row represents an individual time series or gait parameter. The black square indicates the value of the regression coefficient “*β*” for each variable of the linear mixed model. The horizontal line represents the 95% confidence interval estimated by the bootstrap; if the line does not cross the zero line (or large vertical line), it is a reliable estimation.

**Figure A32:**
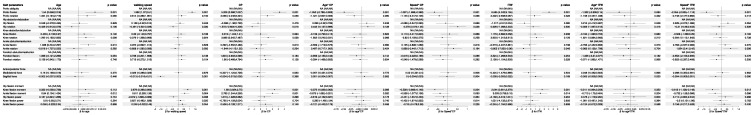
Forest plots showing the effect of age, normalised walking speed, cerebral palsy (CP), idiopathic toe walking (ITW), and the interaction effects between each condition and both age and normalised walking speed on the irreversibility (BDS) of the kinematic and kinetic gait time series for CP, ITW, and healthy children. Each row represents an individual time series or gait parameter. The black square indicates the value of the regression coefficient “*β*” for each variable of the linear mixed model. The horizontal line represents the 95% confidence interval estimated by the bootstrap; if the line does not cross the zero line (or large vertical line), it is a reliable estimation.

**Figure A33:**
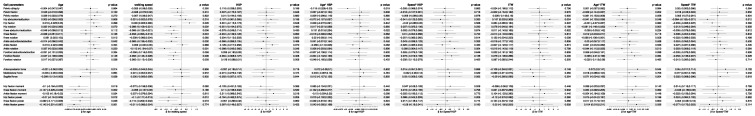
Forest plots showing the effect of age, normalised walking speed, hereditary spastic paraplegia (HSP), idiopathic toe walking (ITW), and the interaction effects between each condition and both age and normalised walking speed on the permutation entropy (PE) of the kinematic and kinetic gait time series for HSP, ITW, and healthy children. Each row represents an individual time series or gait parameter. The black square indicates the value of the regression coefficient “*β*” for each variable of the linear mixed model. The horizontal line represents the 95% confidence interval estimated by the bootstrap; if the line does not cross the zero line (or large vertical line), it is a reliable estimation.

**Figure A34:**
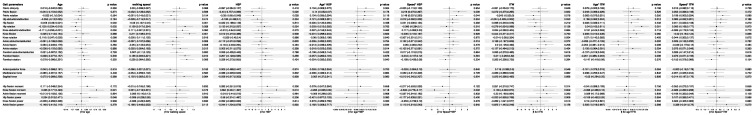
Forest plots showing the effect of age, normalised walking speed, hereditary spastic paraplegia (HSP), idiopathic toe walking (ITW), and the interaction effects between each condition and both age and normalised walking speed on the Shannon’s entropy (SE) of the kinematic and kinetic gait time series for HSP, ITW, and healthy children. Each row represents an individual time series or gait parameter. The black square indicates the value of the regression coefficient “*β*” for each variable of the linear mixed model. The horizontal line represents the 95% confidence interval estimated by the bootstrap; if the line does not cross the zero line (or large vertical line), it is a reliable estimation.

**Figure A35:**
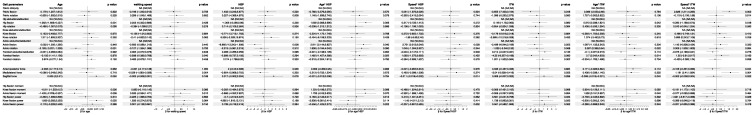
Forest plots showing the effect of age, normalised walking speed, normalised spastic paraplegia (HSP), idiopathic toe walking (ITW), and the interaction effects between each condition and both age and normalised walking speed on the irreversibility (BDS) of the kinematic and kinetic gait time series for HSP, ITW, and healthy children. Each row represents an individual time series or gait parameter. The black square indicates the value of the regression coefficient “*β*” for each variable of the linear mixed model. The horizontal line represents the 95% confidence interval estimated by the bootstrap; if the line does not cross the zero line (or large vertical line), it is a reliable estimation.

Statistical physics metrics vary according to walking speed. The nature and extent of these influences are significantly different in children with HSP, CP, and ITW compared with healthy controls. In healthy children, walking speed [79] exerts a relatively uniform influence on permutation entropy, Shannon’s entropy, and BDS irreversibility. Typically, as walking speed increases, permutation entropy values tend to decrease, indicating a reduction in the randomness of movement. Shannon’s entropy values, conversely, show positive correlations with walking speed, suggesting more complex and varied movement patterns at higher speeds. BDS irreversibility values remain stable, reflecting a consistent and predictable gait pattern irrespective of speed variations. In contrast, children with CP, ITW, and HSP show a more complex and condition-specific relationship between the metrics and walking speed, with speed generally exacerbating the irregularities and unpredictabilities in gait patterns. The interested reader can find specific values in the forest plots of Figure A30, Figure A31, Figure A32, Figure A33, Figure A34 and Figure A35.

In both the Dublin and Madrilenian labs, our analysis reveals that statistical physics metrics remain relatively stable across different age groups in children without gait abnormalities, showing only minimal changes. This fact may reinforce the idea that the maturation of gait patterns in healthy children occurs before the age of inclusion of our subjects (less than 4–5 years) and that later, only a minimal, smooth, and gradual refinement of motor control may occur [80]. The effect of age on abnormal gait varies across different conditions. Age influences statistical physics metrics from proximal (hip and pelvis) and distal joint (ankle) times series in children with CP and HSP, while the impact of age focuses on distal series. Although our study is not longitudinal, we suggest that gait adaptations change in a more global way throughout childhood when corticospinal damage is present.

In our comparison, we observed that statistical physic metrics differ between groups. Interestingly, the Random Forest model using only these measurements of motor control reaches a significant proportion of the classification ability of a powerful technique such as ResNet—see Figure A14 and Figure A15. Our hypothesis is that motor control problems can be as specific for disorders as particular abnormalities in the gait signals. Random Forest based on statistical physics metrics is particularly powerful for distinguishing abnormal from pathological gait, and in general terms, individual kinematic and kinetic joint information is better than including information only from ground reaction force signal. This is an expected observation, as they provide more granular information about gait impairment. The combination of different types of entropies and BDS irreversibility is much better for the classification score than the use of individual parameters. Moreover, the most important features in the Random Forest models are a combination of different types of statistical physics metrics. These facts are suggest that a combination of statistical physics metrics for evaluating gait motor control impairment is needed.

### 4.3. Considerations from a Medical Point of View

Our study has some important implications for the clinical application of machine learning in gait analysis. There are some studies that have incorporated machine learning for patient classification for these disorders [34,81,82], and in general terms, Deep Learning techniques provide good classification scores and different situations. One of the most important drawbacks for the application of Deep Learning (and other machine learning approaches) in clinical practice is the difficult interpretability of these models. For minimising this, we studied the effect of individual time series on classification. For instance, this helps us to understand the relative contribution of the 16 kinematic time series to classification. The ankle and hip flexion time series have a significant role when detecting abnormal vs. normal gait, but when detecting neurological impairment (CP vs. ITW and HSP vs. ITW), knee flexion reveals to be particularly important. This result is in accordance with the previous literature [15,83]. One additional way to understand the behaviour model is the assessment of the optimal resolution of time gait series. Our result support the idea that gait classification highly depends on low-frequency events such as the average value of a particular joint during gait phases instead of particular values at particular critical gait moments (such as toe-off) or minimal or maximum values at particular phases. We believe that this idea is important for the selection of gait features in other typed of approaches. The relatively low impact of sub-windowing the gait cycle on the classification score is also an important aspect in data interpretability. In general terms, classification is resilient to the length and starting point of the sub-windows, which indicates that gait features that are important for distinguishing between gait patterns are pervasive throughout the gait cycle. However, we believe that our results also support that gait cycle strategic sub-windowing could have a role in targeted analysis, enhancing classification performance and the filtering of gait features in the medical applications of machine learning approaches.

A result that is critical to medical applications is the relatively low reliability of models trained in different laboratories. This hampers the direct adaptation of machine learning models trained at other centres. There are several explanations for this finding, such as laboratory setting, differences in data preprocessing, distinct walking distances, and variations in the cohorts. We know that there could exist differences in the cohorts of children with normal gait in different laboratories [84], which have been classically attributed to differences between them but could also represent differences in gait maturation and performance of children from different countries. We also think that our two ITW samples could also have differences; the Madrilenian sample was recruited as part of a clinical study devoted to children with ITW [85], who had been evaluated in a specialised consultation and for whom the need for surgery had been excluded.

The use of statistical physics metrics as outcomes to be incorporated in clinical gait analysis is also an important point to discuss. We believe that this study makes further exploration worthy. We think that the direct use of these metrics will be difficult; yet, their combination through data analysis techniques, even with other metrics of coordination and non-linear analysis, could be interesting for assessing a relatively invisible dimension of gait [33].

## 5. Conclusions

This study aimed to quantify and assess differences between the gait patterns of idiopathic and neurological toe walkers, according to statistical physics-based measures, i.e., Shannon’s entropy, permutation entropy, weighted permutation entropy, and time irreversibility, all reflecting specific dynamics of motor control of the central nervous system. We found that these metrics could effectively distinguish between normal gait and different types of abnormal gait, with the latter exhibiting a more complex and erratic walking pattern, and also among conditions, where disease-specific combinations with walking speed and age reveal different strategies of gait adaptations. Furthermore, the combination of variables improved classification accuracy, which reached levels similar to ResNet, suggesting that metrics could be partially complementary and capturing different facets of complexity in gait kinematic and kinetic time series.

## Figures and Tables

**Table 2:** List of metrics evaluated on the gait time series, along with a synthesis of their statistical physics and gait meaning.

Metric	Statistical Physics Meaning	Gait Meaning
Shannon’s entropy	Uncertainty in the distribution of values	Degree of variability of a gait feature along the gait cycle
Permutation entropy	Uncertainty in the temporal sequence of values	Degree of temporal organisation of a gait feature along the gait cycle without considering the amplitude of changes
Weighted permutation entropy	Uncertainty in the temporal sequence and amplitude	Degree of temporal organisation of a gait feature along the gait cycle considering the amplitude of changes
Irreversibility	Non-linearities, memory	Degree of dependency of a gait feature on a previous value

**Figure A6:**
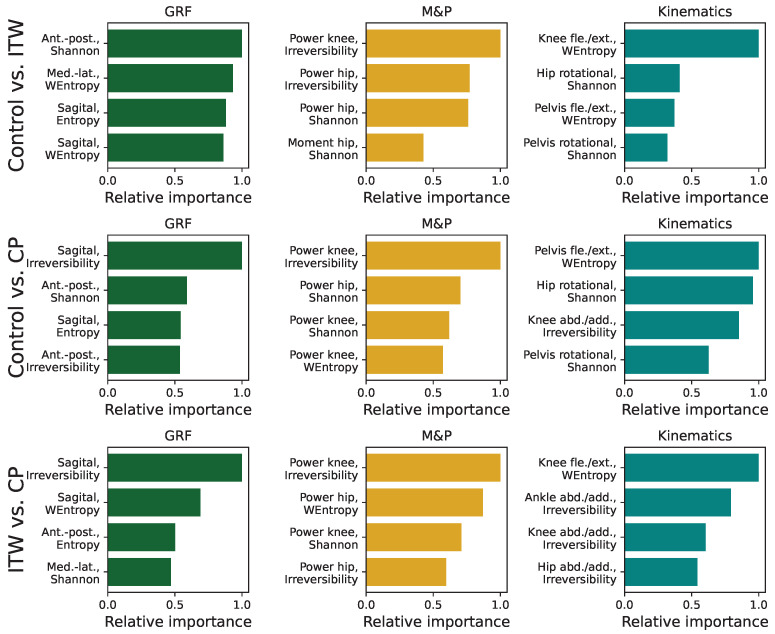
The ranking of the four most important features for Dublin data. *Ant.-post*: anteroposterior; *med.-lat.*: mediolateral; *add./abd.*: adduction–abduction; *fle./ext.*: flexion–extension.

**Figure A7:**
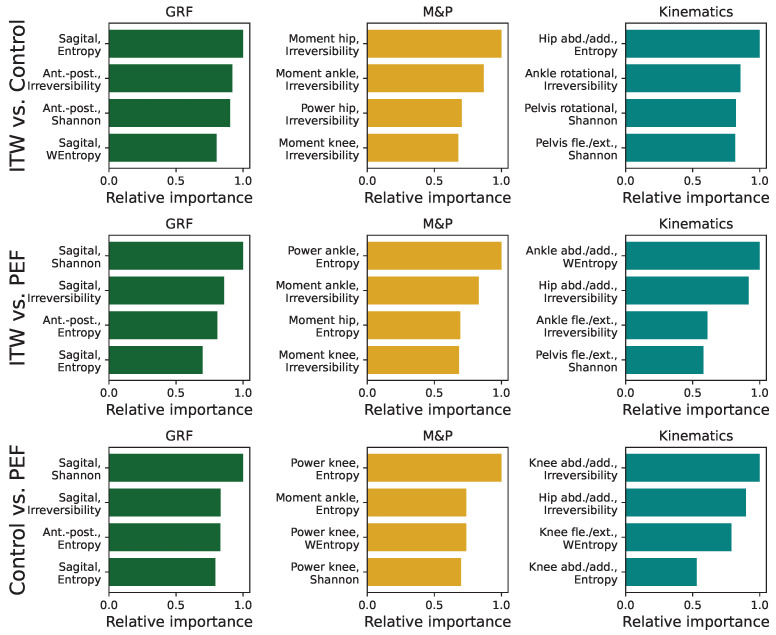
The ranking of the four most important features for Madrilenian data. *Ant.-post*: anteroposterior; *med.-lat.*: mediolateral; *add./abd.*: adduction–abduction; *fle./ext.*: flexion–extension.

## Data Availability

The data presented in this study are available on request from the corresponding authors due to confidentiality issues.
